# Numerical simulation of proppant transport in hydraulic fractures with length-dependent variable aperture

**DOI:** 10.1038/s41598-026-56636-w

**Published:** 2026-06-09

**Authors:** Hui Xiao, Chang Yuan, Chunbing Wang, Guanghui Shi, Qing Wan, Guangda Gao, Mingkang Zhang

**Affiliations:** 1 Chongqing University of Science and Technology, Chongqing, 401331 China; 2 CNPC Greatwall Drilling Company, Beijing, 100101 China; 3 Southwest Oil and Gas Field, Chongqing Branch of China National Petroleum Corporation, Chongqing, 400020 China

**Keywords:** Variable-width fracture, Proppant transport, Euler-Euler, Placement morphology, Energy science and technology, Engineering, Mathematics and computing, Physics

## Abstract

Hydraulic fracturing is vital for enhancing oil and gas production. Proppant transport governs fracture conductivity and is therefore critical. Most studies simplify fractures as constant-width structures, inconsistent with real variable-width fractures.This paper establishes numerical models for proppant transport in variable-width fractures and networks via a solid-liquid two-phase flow method.The results show that, compared to constant-width fractures, variable-width fractures exhibit abrupt changes (reduction) in proppant bank height at width transition points, and the proppant bank trailing edge length is significantly shorter. Proppant particle size has a significant impact: smaller particle sizes increase the leading-edge length by up to 6.7 times and reduce the leading-edge inclination angle by up to 0.22 times. Perforation location greatly influences proppant vortices: when space near the fracture entrance is limited, only a counterclockwise vortex forms near the bottom perforation; if sufficient space exists between the proppant bank and the fracture entrance, besides counterclockwise vortices near the bottom and top perforations, a clockwise vortex forms slightly away from the entrance along the outer edge of the proppant bank; no vortices form in regions far from the fracture entrance. This study provides a new approach for researching proppant transport laws in irregularly shaped fractures.

## Introduction

With the in-depth development of low-permeability and unconventional oil and gas reservoirs, the difficulty of hydrocarbon extraction continues to increase. Hydraulic fracturing technology has become an essential stimulation method^1^. The key to its implementation lies in injecting proppant into the formation to improve seepage channels in the reservoir, thereby enhancing fracture conductivity and increasing hydrocarbon productivity. The proppant placement morphology within the fracture and the interference between branch fractures directly affect fracture conductivity. After fracturing operations, the productivity of the stimulated well is primarily determined by the placement of proppant within the hydraulic fracture. Therefore, understanding the transport laws of proppant within fractures is crucial for the success of reservoir fracturing stimulation^2^.

Experimental research on proppant transport.The research system for proppant transport experiments has progressively evolved from fundamental mechanism exploration to the simulation of complex fracture network environments. In the stage of fundamental settling mechanism research, Stokes^3^ first conducted studies on the free settling of single particles in Newtonian fluids, revealing their basic laws. S.J. Michell et al.^4^ further considered the relationship between particle Reynolds number and the drag coefficient, modified the Stokes formula, and elucidated the settling mechanism of single particles in non-Newtonian fluids. Forevaluating fracturing fluid performance, Larry J. Harrington^5^, Clark^6^, and Kirkby et al.^7^ used concentric cylinder-type transparent testers to systematically study the suspension performance of fracturing fluids like crosslinked gels, clarifying the critical influences of viscosity and shear rate. Cleary et al.^8^ employed more specialized small-scale experimental equipment, revealing the complex effects of wall effects and concentration on settling velocity. As research shifted towards complex fracture environments and engineering parameters, Dayan A et al.^9^ constructed bifurcated and three-dimensional slit models. Zhou Desheng^10^ and Wang J. et al.^11^ used large-scale visual flat-plate fracture apparatus to deeply analyze the influences of large-displacement slickwater fracturing, injection parameters, and proppant combinations in multiple fractures. Gong F. et al.^12^ built a visual static confined fluid settling experimental device, focusing on the settling dynamics of proppants in viscoelastic fluids at low Reynolds numbers, and discovered that fluid elasticity is the key factor causing an exponential decrease in drag. However, the aforementioned experimental studies are mostly based on constant-width parallel plate models, making it difficult to accurately reflect the variable fracture width characteristics of subsurface fractures caused by stress inhomogeneity and rock heterogeneity.

Numerical simulation research on proppant transport.With the improvement in computational power, numerical models have advanced from empirical formulas towards refined multi-physics field coupling. Early research, such as that by Babcock^13^ and R.S. Schols et al.^14^, primarily relied on empirical relationships based on experiments, laying the foundation for numerical simulation. Subsequently, research entered the stage of computational fluid dynamics simulation based on continuum theory, which can be divided into two main methods: The first is the Eulerian-Eulerian Approach, which treats both the fluid and particles as interpenetrating continua. Based on this method, Zhang Tao^15^, Zhang Zheng^16^, and Liu Chunting et al.^17^ simulated the influence of different displacement rates on the proppant bank morphology in constant-width fractures; Oliver Chang et al.^18^ established a mathematical model for quantitatively describing proppant transport behavior in fracture networks based on force analysis on proppants within the fracture and flow distribution in branch fractures; Ren Lan^19^, Zhong Anhai^20^, Liu Chengting^21^, and Zhang Tao et al.^22^ studied the patterns of proppant placement morphology under different fracturing fluid viscosities, sand ratios, and perforation parameters; Hao Lihua^23^, Wang Jie^24^, Luo M.^25^, and Tian Hongzhao et al.^26^ investigated the effects of different fracture structures, fracture angles, widths, and other parameters on proppant placement and transport. The advantage of the Eulerian-Eulerian method lies in its high computational efficiency, making it suitable for engineering-scale problems. The second method is the Eulerian-Lagrangian Approach. The DDPM model used by Hu X. et al.^27^ tracks the particle phase within the continuous fluid phase, enabling a better description of non-equilibrium interactions between particles and fluid, and revealing complex phenomena such as the non-monotonic relationship between proppant concentration and placement height.

To more accurately characterize the mechanical behavior at the particle scale, the Computational Fluid Dynamics-Discrete Element Method (CFD-DEM) Coupling Approach emerged. Zeng Junsheng^28^, Guo Huijuan^29^, and Sun Rui et al.^30^ utilized the CFD-DEM method, which can simulate in detail the interactions between particles, between particles and fluid, and between particles and walls, demonstrating unique advantages in studying transport in intersecting fractures, particle interaction mechanisms, and the role of fibers in channel fracturing. Furthermore, He You’an et al.^31^ adopted the Three-Dimensional Displacement Discontinuity Method, attempting to couple the simulation of proppant transport with the fracture propagation process, representing a development direction towards fully coupled fluid-solid simulation throughout the entire process. Chen Z. et al.^32^ applied the Finite Difference Method to study proppant transport in transverse fractures of horizontal wells. Li J. et al.^33^ conducted an in-depth mechanistic analysis of the placement mechanism in complex fractures, revealing the stages of sand dune deposition and the differences in transport performance of proppants with different particle sizes.

However, considering the excessive workload of this study, the computational demand of using the Computational Fluid Dynamics-Discrete Element Method (CFD-DEM) would be too high. Moreover, this study primarily focuses on the influence of fracture morphology on the overall sand bank morphology, where the transport trajectory of individual proppant particles is not of critical importance. To avoid wasting computational resources, the CFD method was ultimately chosen for the simulation study. All the aforementioned numerical simulations are based on constant-width fracture models, and research on proppant transport under variable-width conditions, considering factors such as pump rate, proppant particle size, and fracturing fluid viscosity, remains limited. Therefore, this study establishes a numerical simulation model for proppant transport in variable-width fractures (wedge-shaped, elliptical, and composite types) to investigate the proppant transport laws in such fractures. A solid-liquid two-phase flow numerical method is adopted to study the proppant transport behavior within variable-width fractures. The numerical model is established, and the transport laws of proppant in variable-width fractures are analyzed through parameters such as the length and inclination angle of the front and rear edges of the sand bank. Furthermore, by incorporating the variable-width fracture morphologies (wedge-shaped, elliptical, composite), the transport characteristics of proppant in a variable-width fracture network are thoroughly studied using the Fluent computational fluid dynamics software on the ANSYS collaborative simulation platform.

## Proppant transport model

### Mathematical model

For solid-liquid two-phase flow of proppant-fracturing fluid, the Euler-Euler two-phase flow numerical method is employed. This method fully considers the coupling between particle phases, the coupling between the particle phase (proppant) and the fluid phase (fracturing fluid), and the turbulent diffusion effect of the particle phase. Continuity and momentum equations are solved separately for the particle phase and the fluid phase.

#### Governing equations

The volume of the liquid phase (fracturing fluid) *V*_*l*_ and the solid phase (proppant) *V*_*s*_ are respectively:

1 $$V_{l} = \int\limits_{V} {\alpha_{l} } dV$$

2 $$V_{S} = \int\limits_{V} {\alpha_{S} } dV$$

Where

3 $$\alpha_{l} + \alpha_{s} = 1$$

In the equations: α——Volume fraction; Subscripts l , s——Represent the liquid phase (fracturing fluid) and solid phase (proppant), respectively.

The continuity equations^28^ for the liquid phase (fracturing fluid) and solid phase (proppant) are respectively:

4 $$\frac{\partial }{\partial t}(\alpha_{l} \rho_{l} ) + \frac{\partial }{{\partial x_{i} }}(\alpha_{l} \rho_{l} U_{li} ) = 0$$

5 $$\frac{\partial }{\partial t}(\alpha_{s} \rho_{s} ) + \frac{\partial }{{\partial x_{i} }}(\alpha_{s} \rho_{s} U_{si} ) = 0$$

Where U——Velocity vector, m/s; α——Volume fraction; t——Injection time, s; ρ——Density, kg/m^3^; **x**——Spatial coordinate, m; x_i_ ——Spatial direction in the i direction, m.

The momentum equations for the liquid phase (fracturing fluid) and solid phase (proppant) are respectively:

6 $$\alpha_{l} \rho_{l} (\frac{{\partial U_{li} }}{\partial t} + U_{lj} \frac{{\partial U_{li} }}{{\partial x_{j} }}) = - \alpha_{l} \frac{{\partial P_{1} }}{{\partial x_{i} }} + \frac{{\partial \Sigma_{lij} }}{{\partial x_{j} }} + I_{li} + \alpha_{l} \rho_{l} g_{i}$$

7 $$\alpha_{s} \rho_{s} (\frac{{\partial U_{si} }}{\partial t} + U_{sj} \frac{{\partial U_{si} }}{{\partial x_{j} }}) = - \alpha_{s} \frac{{\partial P_{2} }}{{\partial x_{i} }} + \frac{{\partial \Sigma_{sij} }}{{\partial x_{j} }} + I_{si} + \alpha_{s} \rho_{s} g_{i}$$

Where ∑——Stress tensor, Pa; I—— Momentum exchange term; P——Pressure, Pa; g——Gravitational acceleration vector, m/s^2^; x_j_——Spatial direction in the j direction, m; U_si_ , U_li_——Velocity components of proppant and fracturing fluid in the i direction, m/s; U_sj_ , U_lj_——Velocity components of proppant and fracturing fluid in the j direction, m/s.

The energy transport equation for the solid phase (proppant) is:

8 $$\frac{3}{2}\rho_{s} [\frac{{\partial \alpha_{s} \Theta_{s} }}{\partial t} + \frac{{\partial \alpha_{s} U_{sj} \Theta_{s} }}{{\partial x_{j} }}] = \frac{\partial }{{\partial x_{i} }}(\kappa_{s} \frac{{\partial \Theta_{s} }}{{\partial x_{i} }}) + \tau_{sij} \frac{{\partial U_{si} }}{{\partial x_{j} }} + \prod\limits_{s} - \alpha_{s} \rho_{s} J_{s}$$

Where K_s_——Energy conduction coefficient; θ_s_——Granular pseudo-temperature, m^2^/s^2^; τ_sij_——Reynolds stress tensor for the solid phase, Pa; J_s_——Energy dissipation due to inelastic collisions, m^2^/s^3^; $\mathop \Pi \limits_{{\mathrm{s}}}$——Energy dissipation due to fluid viscous damping, m^2^/s^3^.

#### Turbulence model

A hybrid turbulence model is used. The liquid phase turbulence model employs the standard k-ε two-equation model^28^:

9 $$\alpha_{l} \rho_{l} [\frac{{\partial k_{l} }}{\partial t} + U_{li} \frac{{\partial k_{l} }}{{\partial x_{j} }}] = \frac{\partial }{{\partial x_{i} }}(\alpha_{l} \frac{{\mu_{l}^{t} }}{{\sigma_{k} }}\frac{{\partial k_{l} }}{{\partial x_{i} }}) + \alpha_{l} \tau_{lij} \frac{{\partial U_{i} }}{{\partial x_{j} }} + \Pi_{kl} - \alpha_{l} \rho_{l} \varepsilon_{l}$$

10 $$\alpha_{l} \rho_{l} [\frac{{\partial \varepsilon_{l} }}{\partial t} + U_{li} \frac{{\partial \varepsilon_{l} }}{{\partial x_{j} }}] = \frac{\partial }{{\partial x_{i} }}(\alpha_{l} \frac{{\mu_{l}^{t} }}{{\sigma_{\varepsilon } }}\frac{{\partial \varepsilon_{l} }}{{\partial x_{i} }}) + \alpha_{l} \frac{{\varepsilon_{l} }}{{k_{l} }} \times \left( {C_{l\varepsilon } \tau_{lij} \frac{{\partial U_{i} }}{{\partial x_{i} }} - \rho_{l} C_{s\varepsilon } \varepsilon_{1} } \right) + \Pi_{\varepsilon l}$$

The solid phase (proppant) turbulence model uses the k_s_-k_ls_ equation^34^

:

11 $$\alpha_{s} \rho_{s} [\frac{{\partial k_{s} }}{\partial t} + \nu_{si} \frac{{\partial k_{s} }}{{\partial x_{j} }}] = \frac{\partial }{{\partial x_{i} }}(\alpha_{s} \rho_{s} K_{s}^{t} \frac{{\partial k_{s} }}{{\partial x_{i} }}) + \alpha_{s} \rho_{s} \tau_{sij} \frac{{\partial U_{si} }}{{\partial x_{j} }} + \Pi_{ks} - \alpha_{s} \rho_{s} \varepsilon_{s}$$

12 $$\alpha_{s} \rho_{s} [\frac{{\partial k_{ls} }}{\partial t} + U_{si} \frac{{\partial k_{ls} }}{{\partial x_{j} }}] = \frac{\partial }{{\partial x_{i} }}(\alpha_{s} \rho_{s} \frac{{v_{ls}^{t} }}{{\sigma_{k} }}\frac{{\partial k_{ls} }}{{\partial x_{i} }}) + \alpha_{s} \rho_{s} \tau_{lsij} \left( {\frac{{\partial U_{si} }}{{\partial x_{j} }} + \frac{{\partial U_{li} }}{{\partial x_{i} }}} \right) + \Pi_{kls} - \alpha_{s} \rho_{s} \varepsilon_{ls}$$

Where K——Turbulent kinetic energy of the continuous phase, m^2^/s^2^; ε——Turbulent dissipation rate, m^2^/s^3^; C_lε_ , C_sε_——Turbulence empirical constants, taken as 1.44 and 1.92, respectively; $\mu_{l}^{t}$ , Π , $K_{s}^{t}$ , $v_{s}^{t}$——Eddy viscosity, turbulent exchange term, shear viscosity, and effective diffusion coefficient, respectively.

#### Constitutive equations

As the governing equations above are not closed, constitutive equations are required for closure. This paper describes the transport and placement laws of proppant-fracturing fluid in variable-width fractures based on the Euler-Euler two-phase flow approach, fully considering solid-solid and solid-liquid coupling effects.

The Gidaspow drag model equation ^34^ describes the interphase state:

13 $$\beta_{ls} = \left\{ {\begin{array}{*{20}l} {\frac{3}{4}C_{D} \frac{{\rho_{l} \alpha_{l} \alpha_{s} \left| {U_{li} - U_{si} } \right|}}{{d_{ps} }}\alpha_{l}^{ - 2.65} } \hfill & {\alpha_{l} > 0.8} \hfill \\ {\frac{{150\alpha_{s} \left( {1 - \alpha_{l} } \right)\mu_{l} }}{{\alpha_{l} d_{ps}^{2} }} + \frac{{1.75\rho_{l} \alpha_{s} \left| {U_{li} - U_{si} } \right|}}{{d_{ps} }}} \hfill & {\alpha_{l} \le 0.8} \hfill \\ \end{array} } \right.$$

Where

14 $$C_{D} = \left\{ {\begin{array}{*{20}l} {\frac{24}{{Re_{s} }}[1 + 0.15(Re_{s} )^{0.687} ]} \hfill & {Re_{s} < 1000} \hfill \\ {0.44} \hfill & {Re_{s} \ge 1000} \hfill \\ \end{array} } \right.$$

15 $$Re_{s} = \frac{{\rho_{l} d_{ps} \alpha_{l} |U_{si} - U_{li} |}}{{\mu_{l} }}$$

Where C_D_——Drag coefficient for a single particle, determined based on Reynolds number; Re_s_——Particle Reynolds number; d_ps_——Particle diameter.

The granular temperature is proportional to the kinetic energy associated with the random motion of particles. The transport equation derived from kinetic theory is as follows:

16 $$\frac{3}{2}\rho_{s} [\frac{\partial }{\partial t}(\alpha_{s} \Theta_{s} ) + \nabla \cdot(\alpha_{s} u_{s} \Theta_{s} )] = \nabla \cdot(k_{s} \nabla \Theta_{s} ) + \tau_{s} :\nabla u_{s} - J_{s} + \Pi_{\Theta }$$

Where θ_s_——Granular pseudo-temperature, m^2^/s^2^; K_s_——Granular energy diffusion coefficient, kg/(m·s); J_s_——Energy generated by inelastic collisions, kg/(m·s^3^); Π_θ_——Energy dissipation due to fluid viscous damping, kg/(m·s^3^).

#### Boundary conditions

Boundary conditions include inlet, outlet, and wall boundaries. The inlet boundary condition is set as a velocity inlet, and the outlet boundary condition is set as a pressure outlet. At the wall, the tangential velocity and temperature of the solid phase are solved using the Johnson-Jackson^35^ model.

17 $$W_{E} = \frac{{W_{1} L_{1} }}{{\frac{1}{2}\Pi W_{2} L_{2} + W_{1 - 1} L_{1 - 1} }}$$

18 $$k_{{\mathrm{s}}} \frac{{\partial \Theta_{{\mathrm{s}}} }}{\partial x} = \frac{{\phi \pi \left| {{{\boldsymbol{\upnu}}}_{{{\mathrm{sl}}}} } \right|\rho_{{\mathrm{s}}} \alpha_{{\mathrm{s}}} g\sqrt {\Theta_{{\mathrm{s}}} } }}{{2\sqrt 3 \alpha_{{\mathrm{s}}}^{{{\mathrm{max}}}} }} - \frac{{\sqrt 3 \pi \rho_{{\mathrm{s}}} \alpha_{{\mathrm{s}}} g\left( {1 - e_{{\mathrm{W}}}^{2} } \right)\sqrt {\Theta_{{\mathrm{s}}} } }}{{4\alpha_{{\mathrm{s}}}^{{{\mathrm{max}}}} }}\Theta_{{\mathrm{s}}}$$

Where V_ls_——Slip velocity vector between solid phase and wall, m/s; τk、τf——Shear stress tensor components at the wall due to solid kinetic collision and friction, Pa; w——Inward normal vector to the wall; Φ——Specularity coefficient, taken as 0.001; δw——Wall friction angle, taken as π/10; e_w_——Coefficient of restitution for particle-wall collisions, taken as 0.9.

### Geometric model and validation

#### Geometric model

**Fig. 1:**
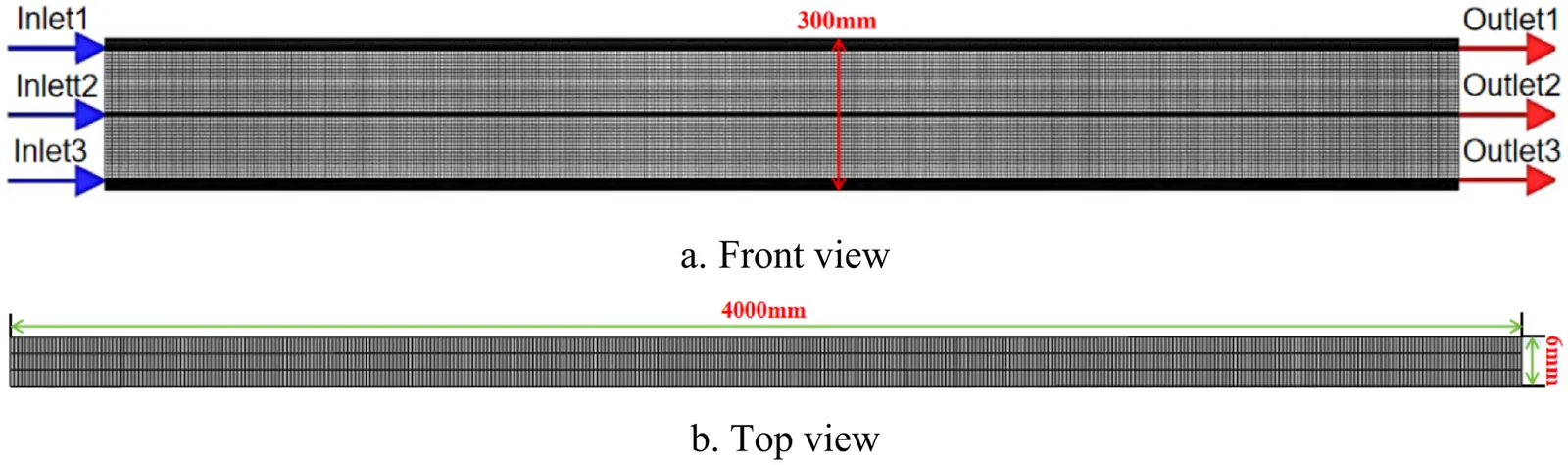
3D vertically rectangular constant-width single fracture model after meshing.

SpaceClaim 3D modeling software was used to establish a vertical rectangular constant-width single fracture model with dimensions of 4000mm × 300mm × 6mm. Three inlets and three outlets were set up. Leveraging the advantages of hexahedral structured meshes in terms of flow direction orthogonality, regularity of spatial node arrangement, and numerical calculation accuracy, the hexahedral structured meshing method was selected based on the geometric characteristics of the 3D model. A grid independence study comparing results showed that when the mesh count increased from 83,564 to 102,358, the resulting error was 2.1%. This indicates that the calculation results fall within the acceptable error range. Considering computational cost, this study selected the mesh configuration with 83,564 elements. The 3D model with the structured mesh applied is shown in Fig. 1.

#### Proppant transport experiment

**Fig. 2:**
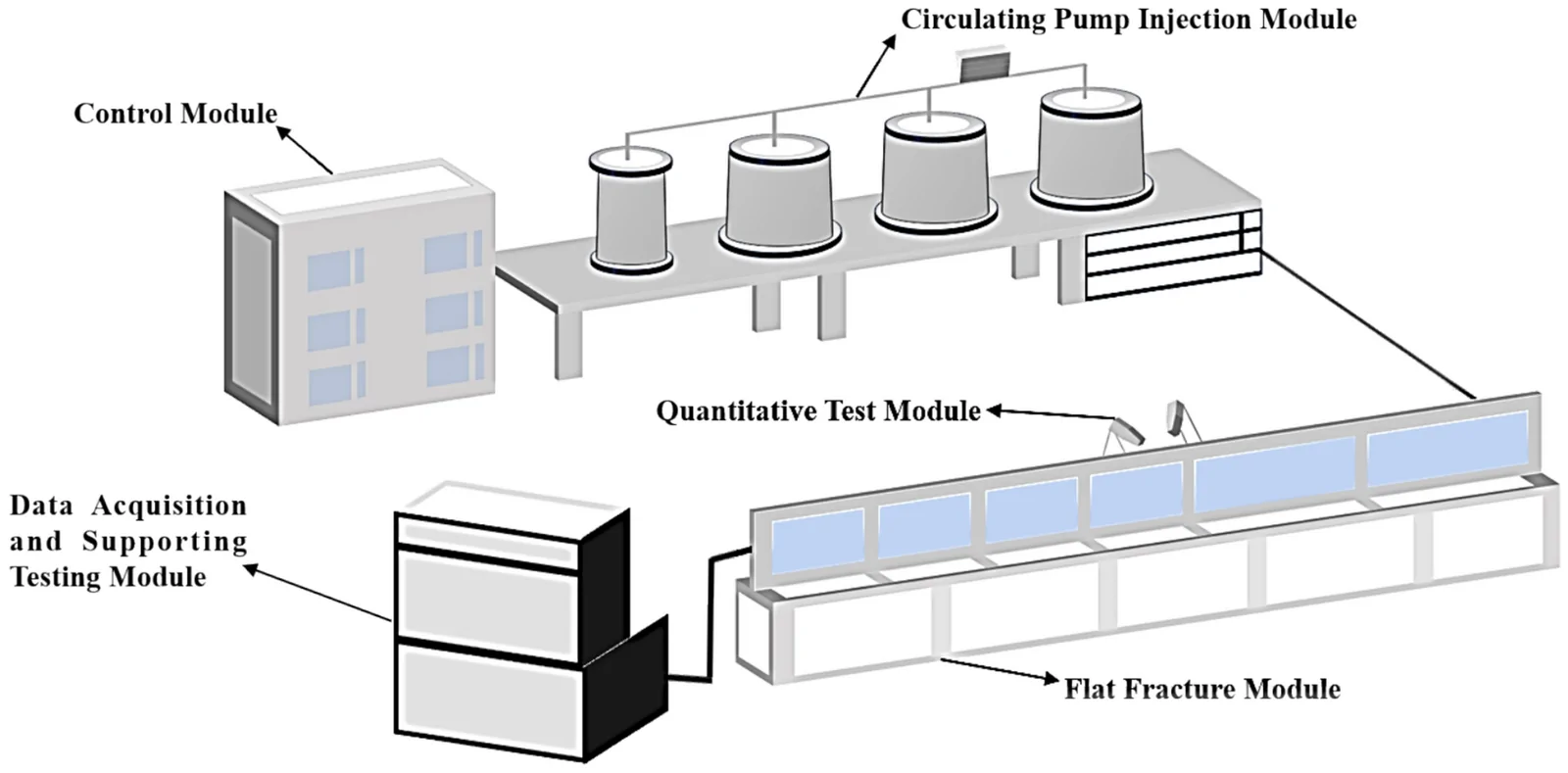
Schematic diagram of multi-scale proppant delivery physics simulation system.

The experiment utilized a visualized flat-plate proppant transport and placement apparatus, also known as a multi-scale proppant delivery physical simulation system. This system consists of five main modules: control module, flat-plate fracture module, quantitative testing module, circulation pumping module, and data acquisition and auxiliary testing module. The fracture in the experimental device was made of high-strength transparent organic glass, simulating a constant-width rectangular fracture. The fracture dimensions were 4000mm × 300mm × 6mm, identical to the setup in Fig. 1, with three inlets and three outlets simulating three perforation holes at the upper, middle, and lower positions of the fracture entrance. Figure 2 shows a schematic diagram of the multi-scale proppant delivery physical simulation system.

According to the Reynolds similarity criterion, the Reynolds number in the formation fracture and that in the laboratory flat-plate fracture must satisfy:

19 $$Re_{m} = Re_{n} \Leftrightarrow \frac{{\rho_{m} d_{m} \nu_{m} }}{{\mu_{m} }} = \frac{{\rho_{n} d_{n} \nu_{n} }}{{\mu_{n} }}$$

Where Re——Reynolds number(dimensionless; subscripts m and n denote laboratory experiment and field operation conditions, respectively); v——velocity, m/s; ρ——fluid density, kg/m^3^;μ——fluid viscosity, Pa·s.

The characteristic length d is calculated by:

20 $$d = \frac{2hw}{{h + w}}$$

Where h——fracture height, m; w——fracture width, m.

To ensure that the flow state in the laboratory simulation experiment is similar to that in the formation fracture during actual field fracturing, the physical properties of the fluid used in the laboratory experiment are kept consistent with those used in the field. Based on the structural parameters of the laboratory setup and the geometric parameters of the actual field fracture, Eqs. (19) and (20) can be transformed into:

21 $$\nu_{m} d_{m} = \nu_{n} d_{n} \Leftrightarrow Q_{m} = \frac{0.042}{{h_{n} + 0.01}}Q_{n}$$

Where Qm——laboratory flow rate, L/min; Qn——field flow rate, L/min; hn——actual formation fracture height, m

Using the above conversion, when the field fracturing flow rate is 18–20 m^3^/min and the fracture height is 40–50 m, the equivalent laboratory flow rate ranges from approximately 15 to 21 L/min. Based on this calculation, the laboratory flow rate is set to 18 L/min. The experimental parameters are listed in Table 1.

**Table 1:** Experimental parameters for constant-width fracture at different pump rates.

Pumping rate (L/min)	Proppant size(mesh)	Proppant concentration(%)	Fracturing fluid viscosity(mPa·s)	Fracture width(mm)
18	40/70	15	2.5	6

Since field construction typically uses high-volume (18–20 m^3^/min) pumping of fracturing fluid, an 18 L/min flow rate was selected for the experimental conditions, with other parameters also set based on field conditions. The experimental parameters are shown in Table 1, which lists the experimental parameters for different pumping flow rates under equal-width fractures.

#### Validation and comparison

To verify the correctness of the established proppant-fracturing fluid two-phase flow numerical model, results were compared with laboratory flat-plate proppant transport experiments. Based on the correctness of the model validation, parameters such as the drag coefficient in the mathematical model were calibrated. Since the numerical simulation inlet boundary condition is a velocity inlet, the flow rate used in the laboratory experiment needed to be converted to inlet velocity using the following formula. Conversion results are shown in Table 2:

22 $$V = \frac{Q}{H \times W}$$

Where V——Inlet velocity in the numerical model, m/s; Q——Laboratory experiment flow rate, m^3^/s; H——Length of perforation along the fracture height direction, m; W——Fracture width, m.

**Table 2:** Inlet velocity conversion results.

Experimental pumping rate (L/min)	H(mm)	W(mm)	*v*(m/s)
18	30	6	1.67

**Fig. 3:**
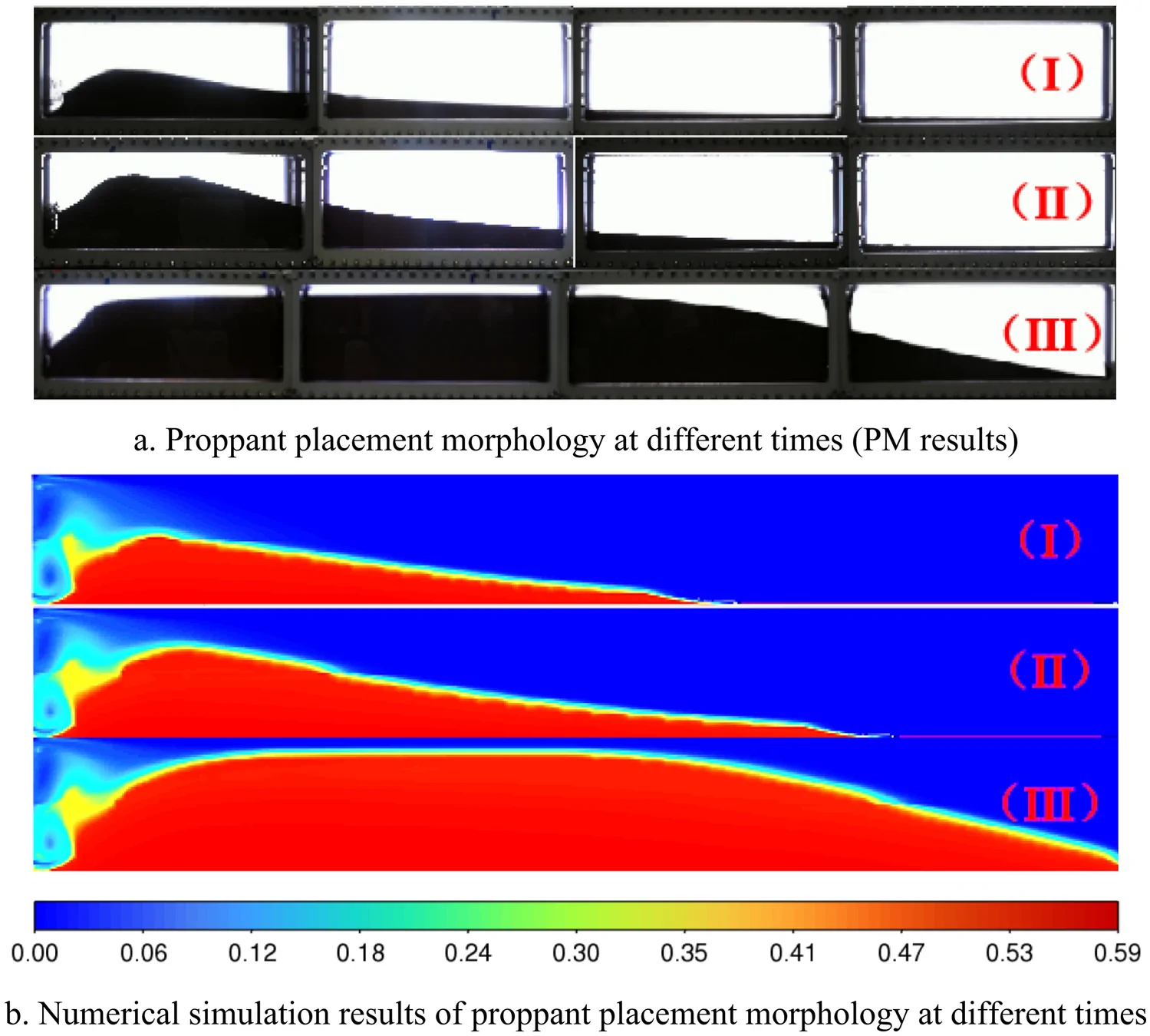
Comparison of numerical simulation (NS) and physical model (PM) results.

**Fig. 4:**
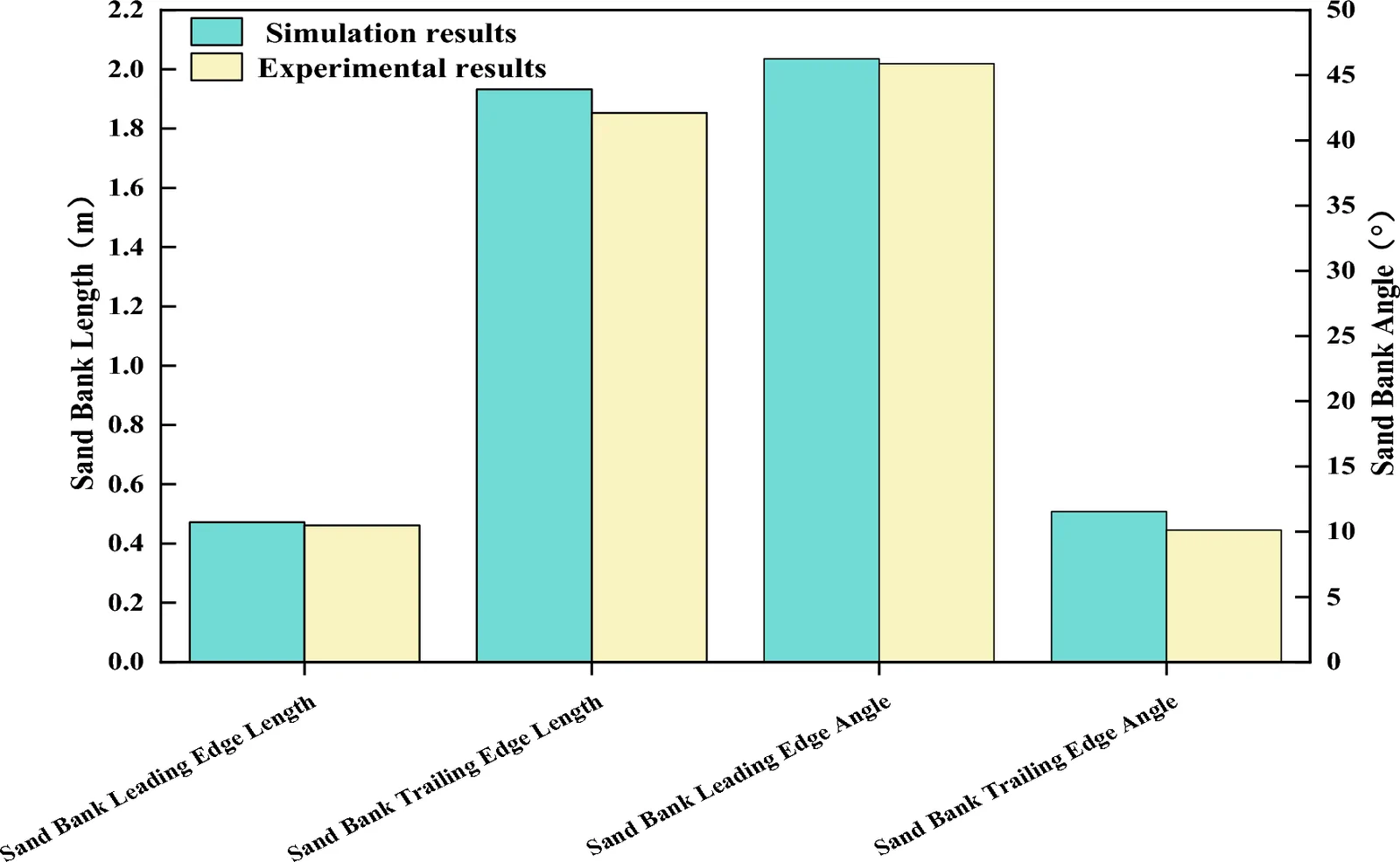
Comparison of physical model test results and numerical simulation results.

By comparing the results of physical model experiments and numerical simulations, as shown in Fig. 3, it can be observed that the sand embankment morphology at each time point in the physical model experiments and numerical simulations are highly consistent. As shown in Fig. 4, the parameter comparison results between the physical model experiments and numerical simulations are similar, with a similarity of over 90%, and an average similarity of 92.98%. However, there are some differences in the degree of unfilled areas near the wellbore. The degree of unfilledness in the simulation results is higher than that in the physical model experiment results. This deviation stems from the prediction error in proppant drag force caused by complex fluid-solid coupling effects within the plate crack under experimental conditions. Given the sensitivity characteristics of the drag coefficient model in particle settling dynamics, the sand distribution at the crack front shown in this numerical simulation is acceptable.

### Proppant transport model for fractures with varying widths

#### Establishment and meshing of single variable-width fracture models

The idealized constant-width rectangular fracture model differs significantly from reality. It is necessary to design variable-width (wedge-shaped, elliptical, composite) fracture models that better conform to actual conditions to improve understanding of proppant transport laws within them.

This paper uses SpaceClaim 3D modeling software to establish geometric models for single variable-width fractures. Specific geometric parameters for the numerical models are: inlet fracture width 10mm, outlet fracture width 2mm, fracture length 4000mm, number of fracture segments 5.

**Fig. 5:**
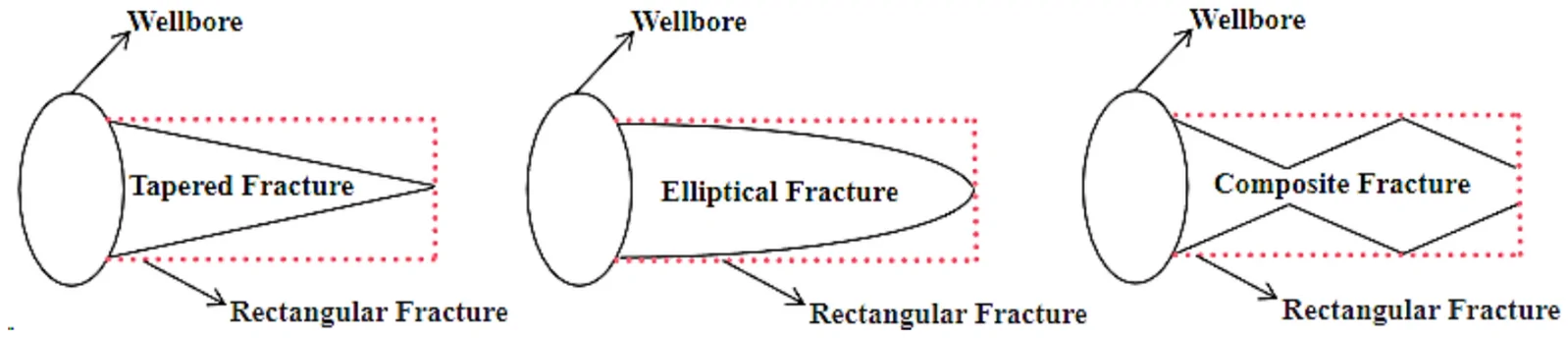
Schematic top view of variable-width fractures (wedge-shaped, elliptical, composite).

When constructing the variable-width (wedge-shaped, elliptical, composite) fracture models (Fig. 5), equivalent calculations of fracture length and width were performed for comparison between different morphologies. The variable-width fracture length is defined as the perpendicular distance between the fracture outlet and inlet. The equivalent width formulas for wedge-shaped and elliptical variable-width fractures are expressed as:

23 $$W_{{\mathrm{w}}} = \frac{{W_{1} L_{1} }}{{\frac{1}{2}(W_{2} + W_{3} )L_{3} }}$$

24 $$W_{E} = \frac{{W_{1} L_{1} }}{{\frac{1}{2}\Pi W_{2} L_{2} + W_{1 - 1} L_{1 - 1} }}$$

Where W_w_, W_E_——Equivalent fracture width, mm; L_1_ , L_2_ , L_1-1_——Length of constant-width fracture, length of variable-width fracture, length of rectangular section within elliptical variable-width fracture, mm; W_1_ , W_2_ , W_3_ , W_1-1_——Inlet/outlet width of constant-width fracture, inlet width of variable-width fracture, outlet width of variable-width fracture, width of rectangular section within elliptical variable-width fracture, mm.

Geometric model parameters and schematic diagrams for each fracture morphology are shown in Tables 3 and 4.

**Table 3:** Geometric model parameters for single variable-width fractures.

Fracture patterns	Inlet fracture width (mm)	Outlet fracture width (mm)	Equivalent fracture width (mm)	Fracture length (mm)	Number of stages (stages)	Stage fracture length (mm)	Model number
Tapered fracture	10	2	6	4000	5	800	①
Elliptical fracture	7.1	2	6	4000	5	800	②

**Table 4:**
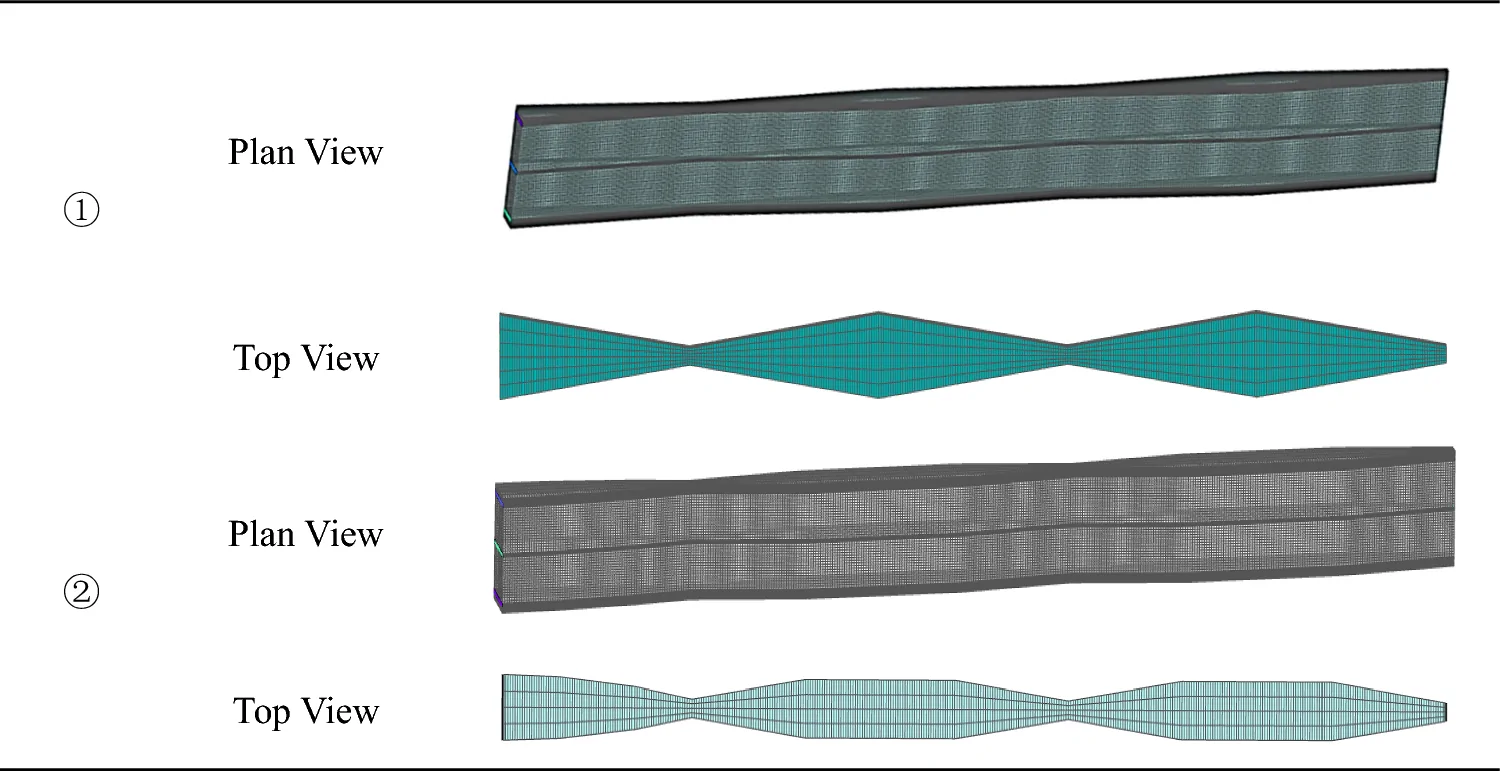
Schematic diagrams of single variable-width fracture models.

Composite fracture models are shown in Tables 5 and 6. Among them, Composite Fracture (I) consists of elliptical + tapered + elliptical + elliptical + elliptical; Composite Fracture (II) consists of elliptical + tapered + elliptical + tapered + elliptical; Composite Fracture (III) consists of elliptical + tapered + tapered + tapered + elliptical.

**Table 5:** Geometric model parameters for composite variable-width fractures.

Fracture patterns	Inlet fracture width (mm)	Outlet fracture width (mm)	Equivalent fracture width (mm)	Fracture length (mm)	Number of stages (stages)	Stage fracture length (mn)	Model number
Composite fracture (Ⅰ)	8.56	2	6	4000	5	800	①
Composite fracture (II)	8.56	2	6	4000	5	800	②
Composite fracture (III)	8.56	2	6	4000	5	800	③

**Table 6:**
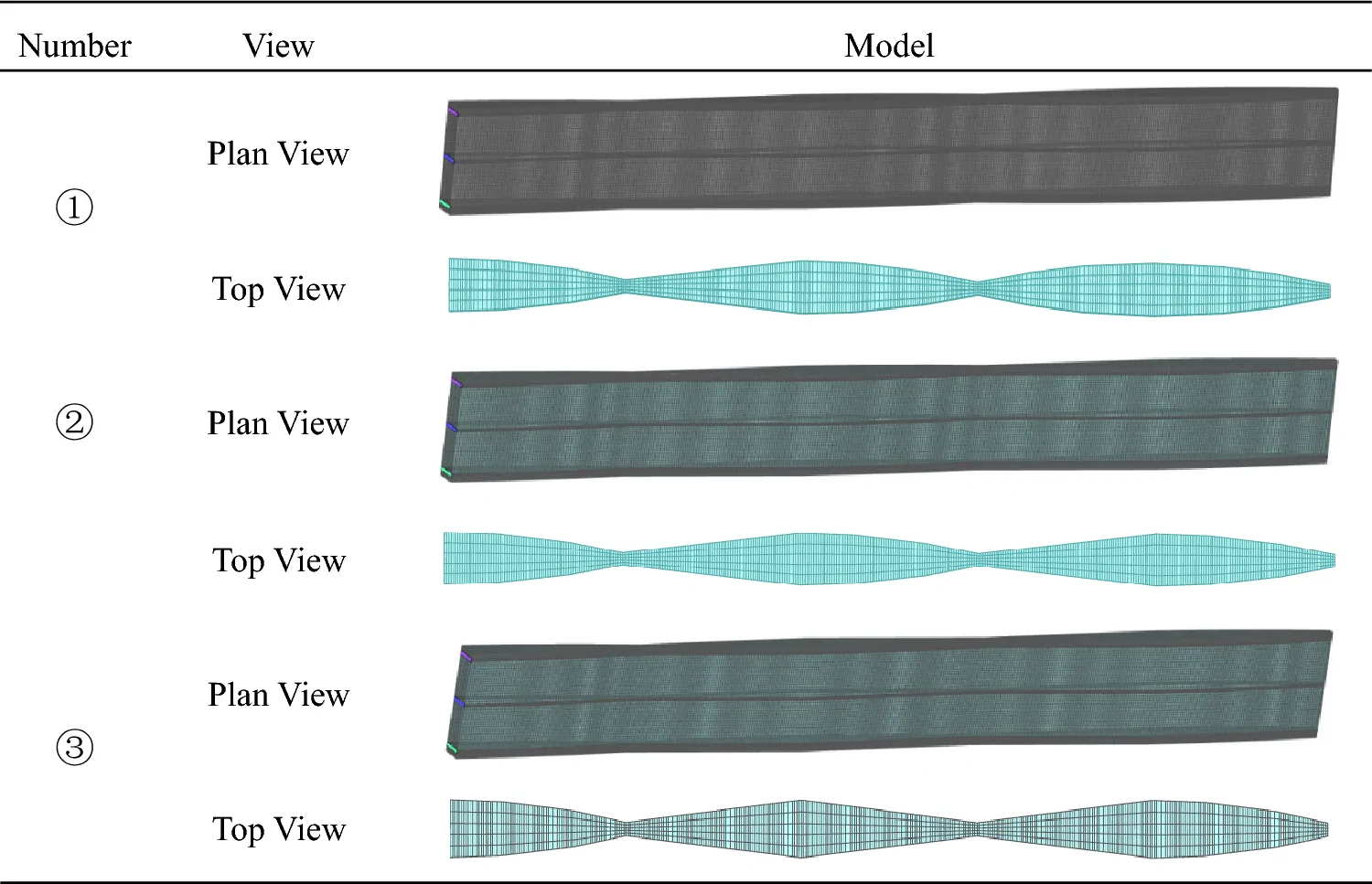
Schematic diagrams of composite variable-width fracture models.

#### Establishment and meshing of variable-width fracture network model

Model geometric parameters are set as follows:

1. Main Fracture Structure: Gradually varying width (inlet 10mm, outlet 2mm), length: 3000mm, segments: 3;
2. Primary Branch Fracture Structure: Gradually varying width (inlet 2mm, outlet 1mm), length: 2000mm, segments: 3;
3. Secondary Branch Fracture Structure: Gradually varying width (inlet 2mm, outlet 1mm), length: 666mm, segments: 1 (total).

Fracture network parameters and model schematics are shown in Tables 7 and 8.

**Table 7:** Geometric model parameters for variable-width fracture network.

Main fracture segment number (segments)	First branch fracture segment number (segment)	Second branch fracture segment number (segment)	Main fracture equivalent fracture width (mm)	Branch fracture equivalent fracture width (mm)
3	3	1	6	1.5

**Table 8:**
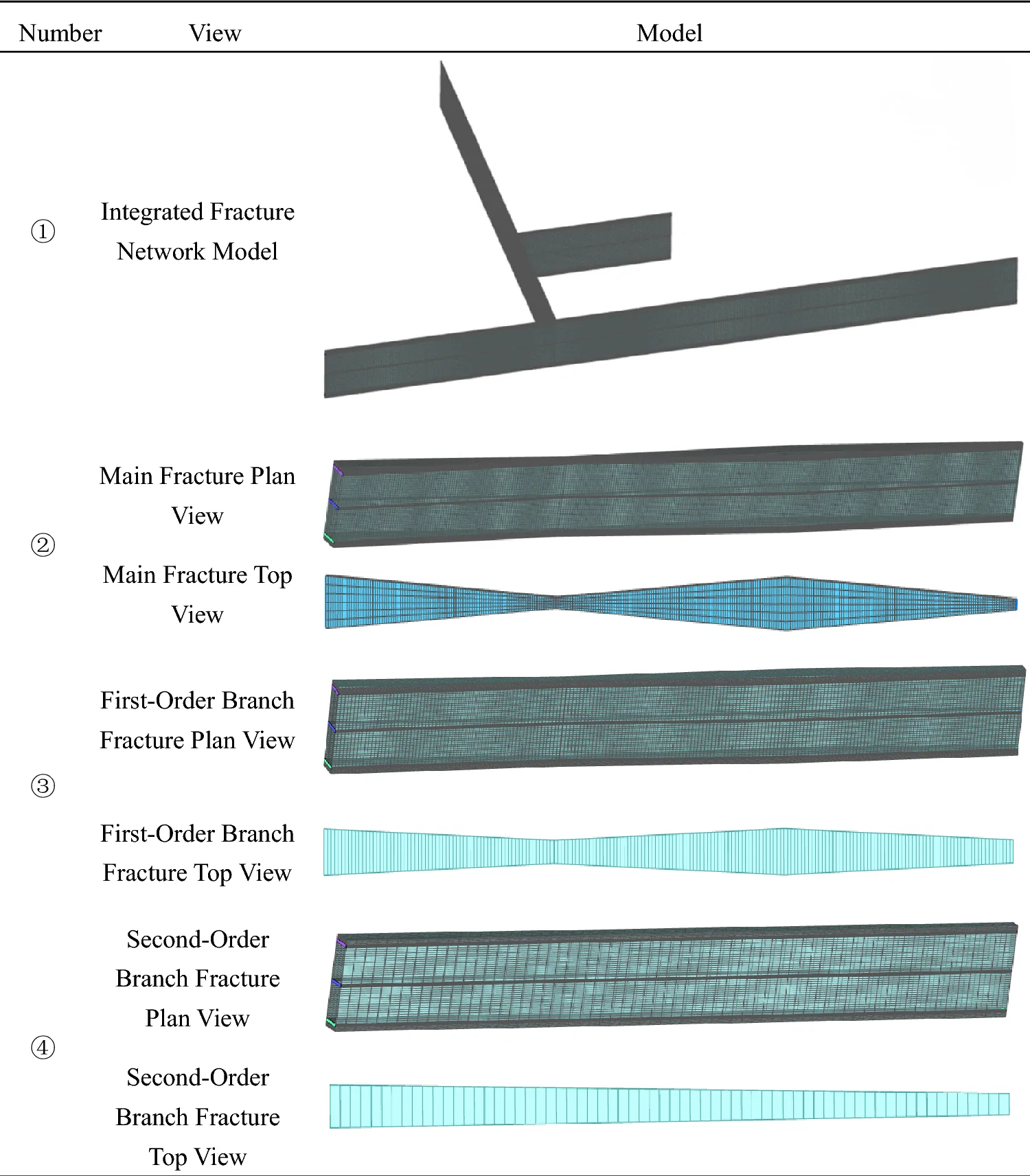
Variable-width fracture network model diagrams.

## Results and discussion

### Transport laws in different fracture morphologies

#### Pump rate

For the three pumping rates of 6 L/min, 18 L/min, and 30 L/min, the proppant mass was set to 9 kg, the inlet velocity to 1.67 m/s, and the pumping durations to 225 s, 75 s, and 45 s, respectively. Under the above conditions, the proppant placement morphology in constant-width, wedge-shaped, elliptical, and composite fractures was simulated. Comparison results of placement morphology are shown in Table 9.

**Table 9:**
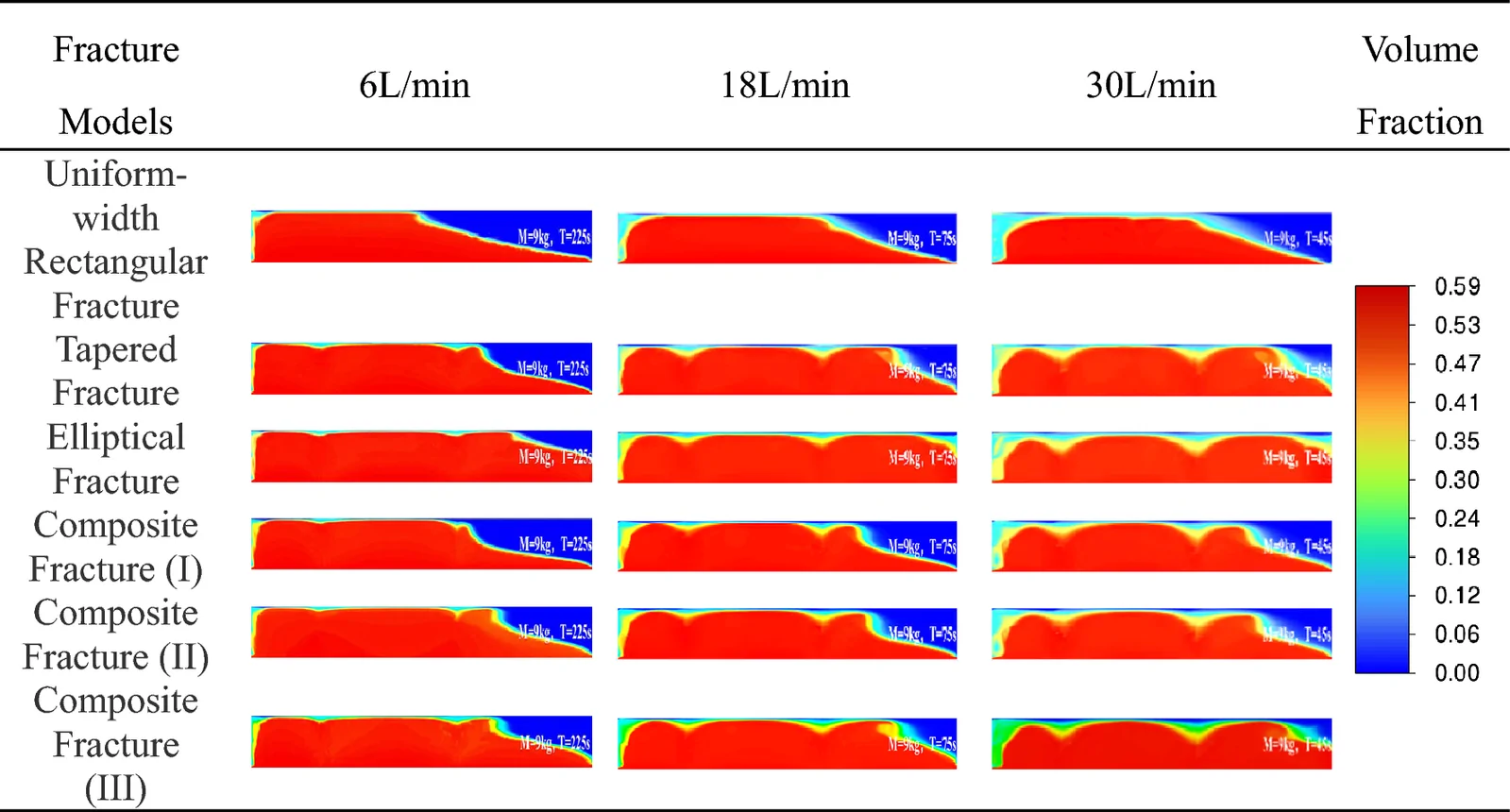
Proppant distribution morphology within fractures at different pump rates.

**Fig. 6:**
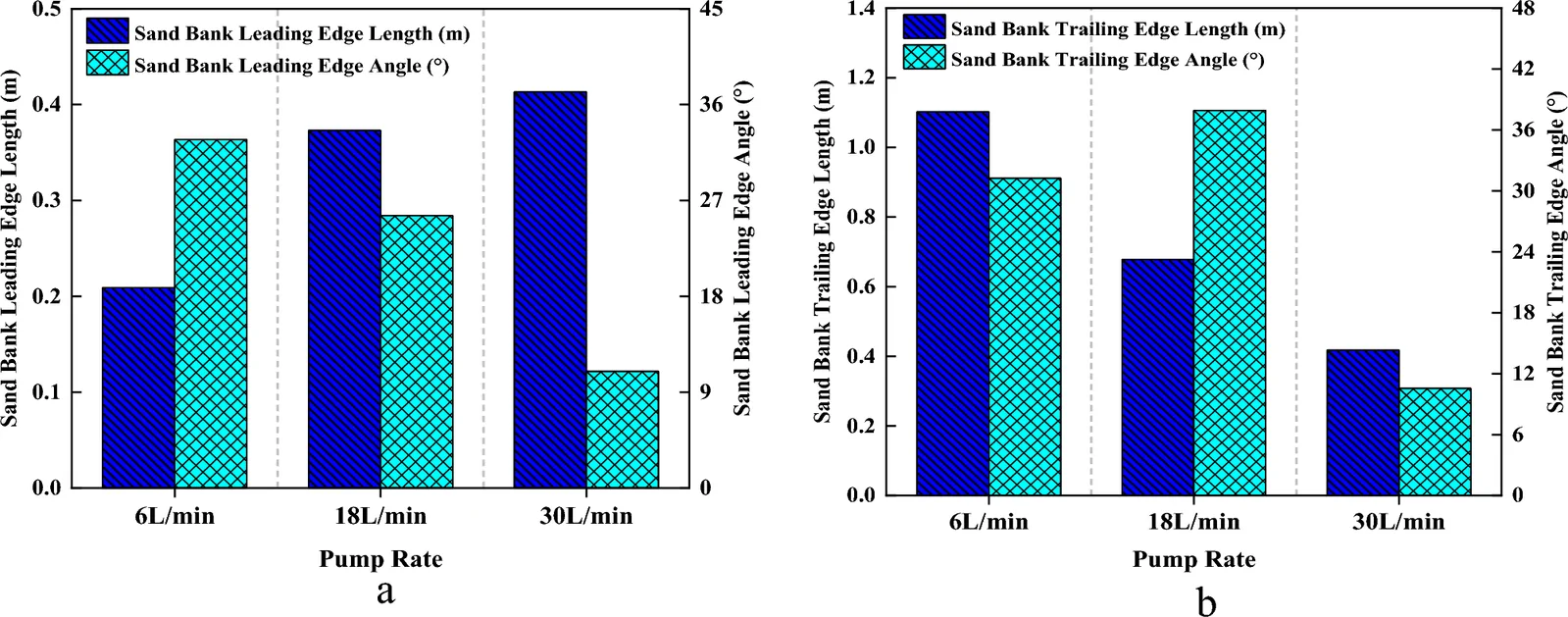
Relationship between characteristic parameters and pump rate (**a**) Proppant Leading Edge (**b**) Trailing Edge of Proppant.

As the pump rate increases, the proppant bank within the fracture migrates further towards the fracture tip. The leading-edge length of the proppant bank increases, the ineffective support area near the wellbore expands continuously, and the trailing-edge length decreases continuously. The main body length of the proppant bank increases, and the equilibrium height decreases. The trailing-edge length in variable-width fractures is shorter than in constant-width fractures, with the elliptical fracture having the shortest trailing-edge length, indicating proppant migrates farthest in elliptical fractures. Analysis suggests that narrow sections of the fracture alter the flow state of the proppant, and fracture morphology also affects proppant bank placement. The relationship between characteristic parameters and pump rate (Fig. 6) shows that as flow rate increases, the proppant bank leading-edge length increases, the leading-edge inclination angle decreases, the proppant bank migrates further towards the fracture tip, causing the trailing-edge length to decrease and the trailing-edge inclination angle to increase.

**Fig. 7:**
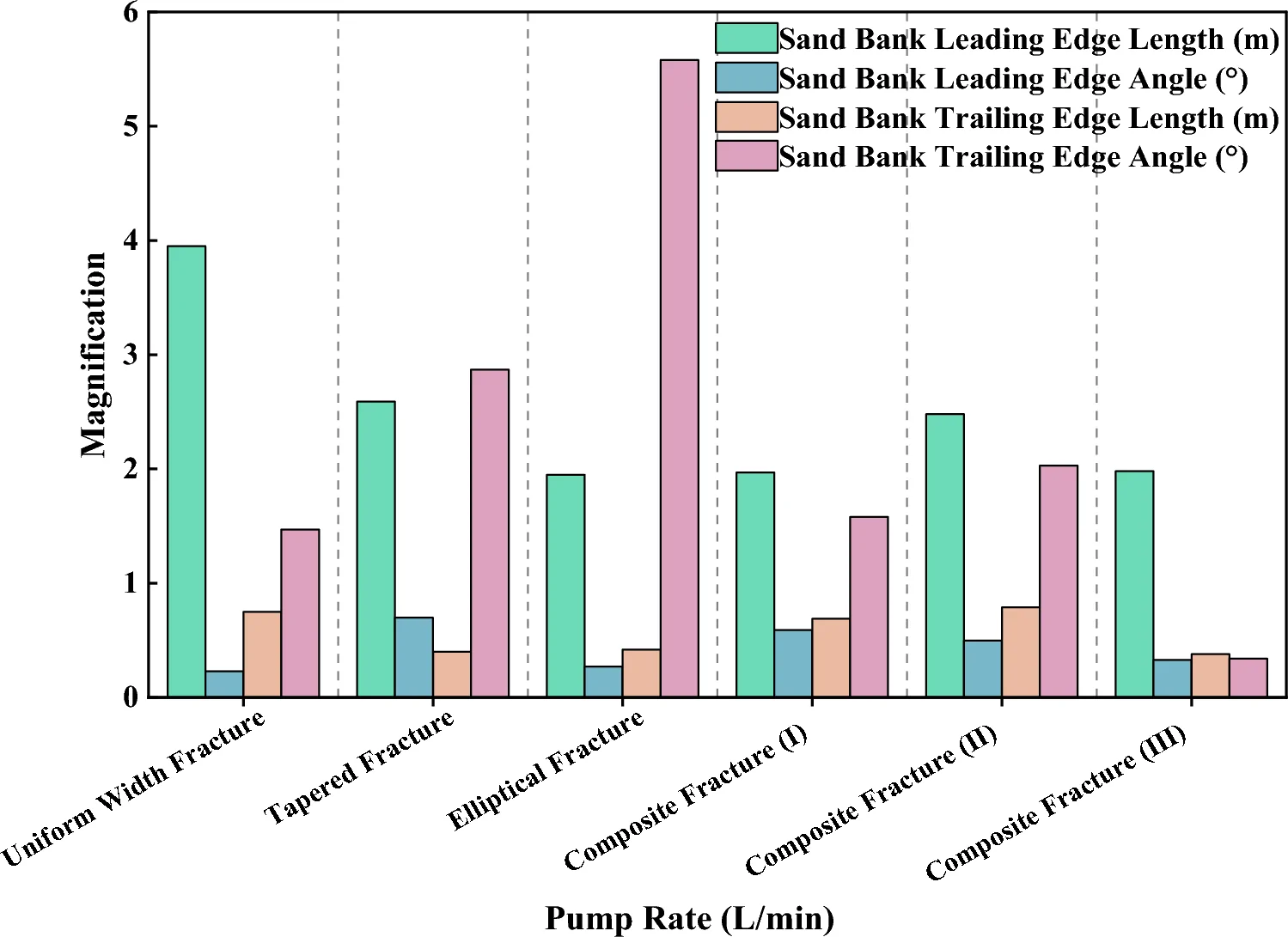
Trend of various parameters for different fracture types with pump rate.

Taking the change from 6 L/min to 30 L/min as the study object. Figure 7 shows the trend of parameters for each fracture type with flow rate. Flow rate has a significant impact on proppant bank leading-edge length and trailing-edge inclination angle. The constant-width fracture exhibits the largest increase in leading-edge length (3.95 times). The elliptical fracture shows the largest increase in trailing-edge inclination angle (5.58 times). Composite fracture (III) shows the largest decrease in trailing-edge inclination angle (reducing to 0.48 times the original angle).

**Fig. 8:**
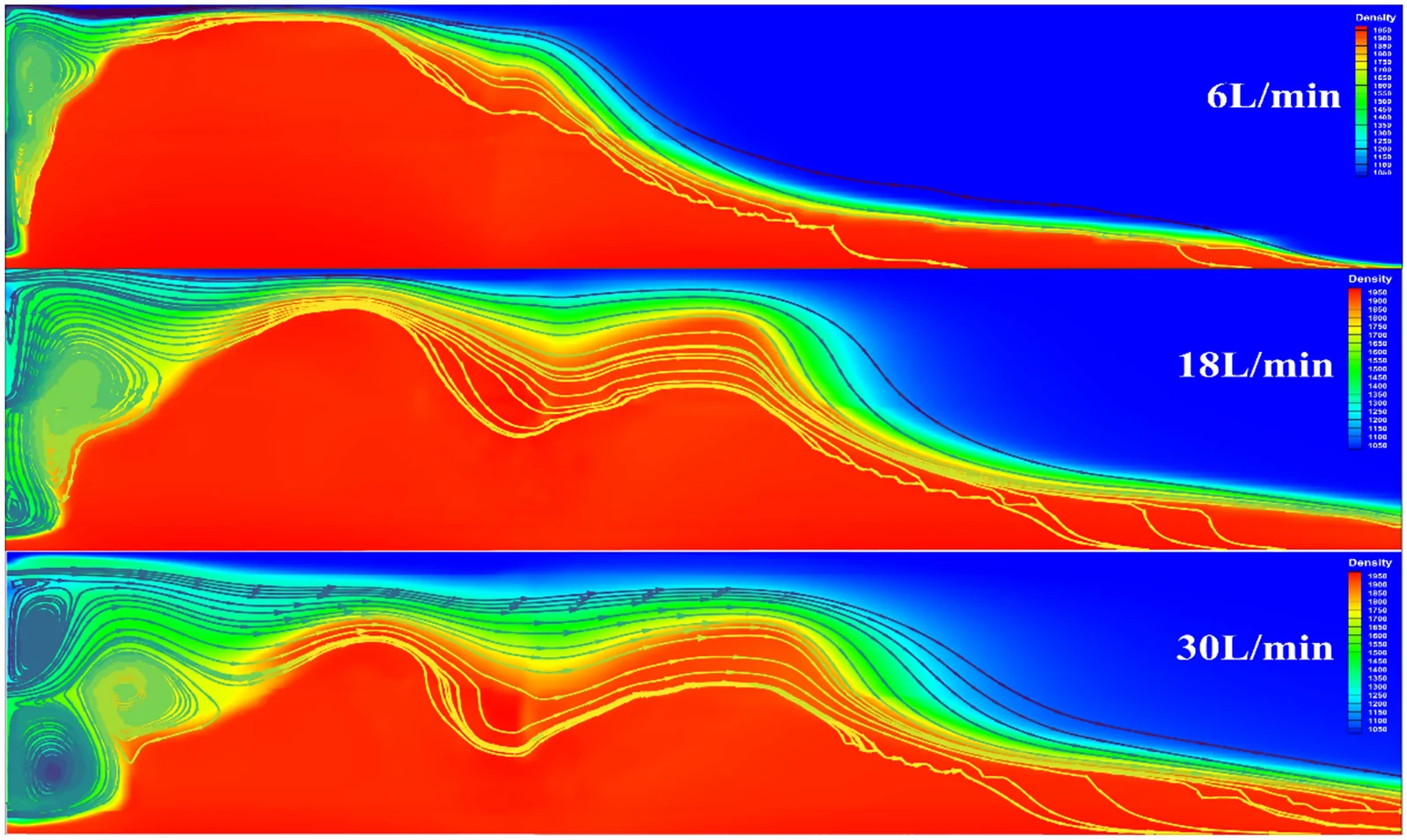
Slurry pathlines within Composite (III) variable-width fracture at different pump rates.

Slurry pathlines of sand-carrying fluid at different discharges in the composite (Ⅲ) variable seam width fracture with sand injection volume M=9kg and pumping time of 25s, as shown in Fig. 8. Trace diagram shows the flow trajectory of the sand mixing fluid within the fracture. As flow rate increases, the vortex morphology and flow direction change. At 30 L/min, the vortex formed near inlet 3 rotates counterclockwise. When sufficient space exists ahead of the proppant bank, the lower vortex merges with slurry from inlet 2, encountering the bank and forming a clockwise vortex slightly ahead. At inlet 1, a clockwise vortex forms. At 18 L/min, reduced space ahead of the bank limits the counterclockwise vortex formation near inlet 3, but a larger clockwise vortex still forms centrally. At 6 L/min, significant proppant accumulation near the inlet severely restricts vortex formation, allowing only a clockwise vortex similar to the unfilled zone near the inlet. Vortices only appear near the entrance region; flow is plug-like elsewhere, with noticeable fluctuations at fracture width transition points.

#### Fracturing fluid viscosity

For the three fracturing fluid viscosities of 2.5 mPa·s, 5 mPa·s, and 10 mPa·s, the proppant mass was set to 9 kg, the pumping rate to 18 L/min, and the pumping duration to 75 s. Under the above conditions, the proppant placement morphology in constant-width, wedge-shaped, elliptical, and composite fractures was simulated. Comparison results are shown in Table 10.

**Table 10:**
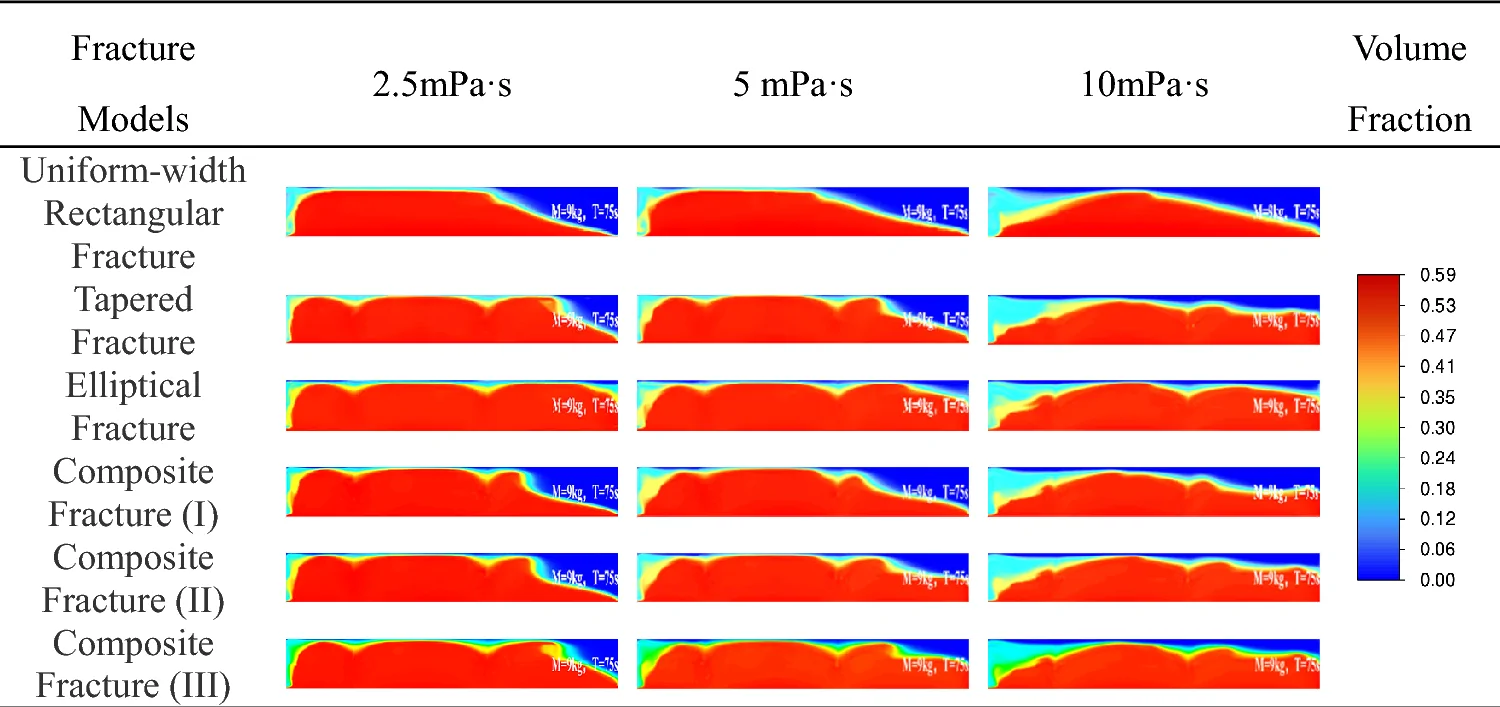
Proppant distribution morphology within fractures at different fracturing fluid viscosities.

**Fig. 9:**
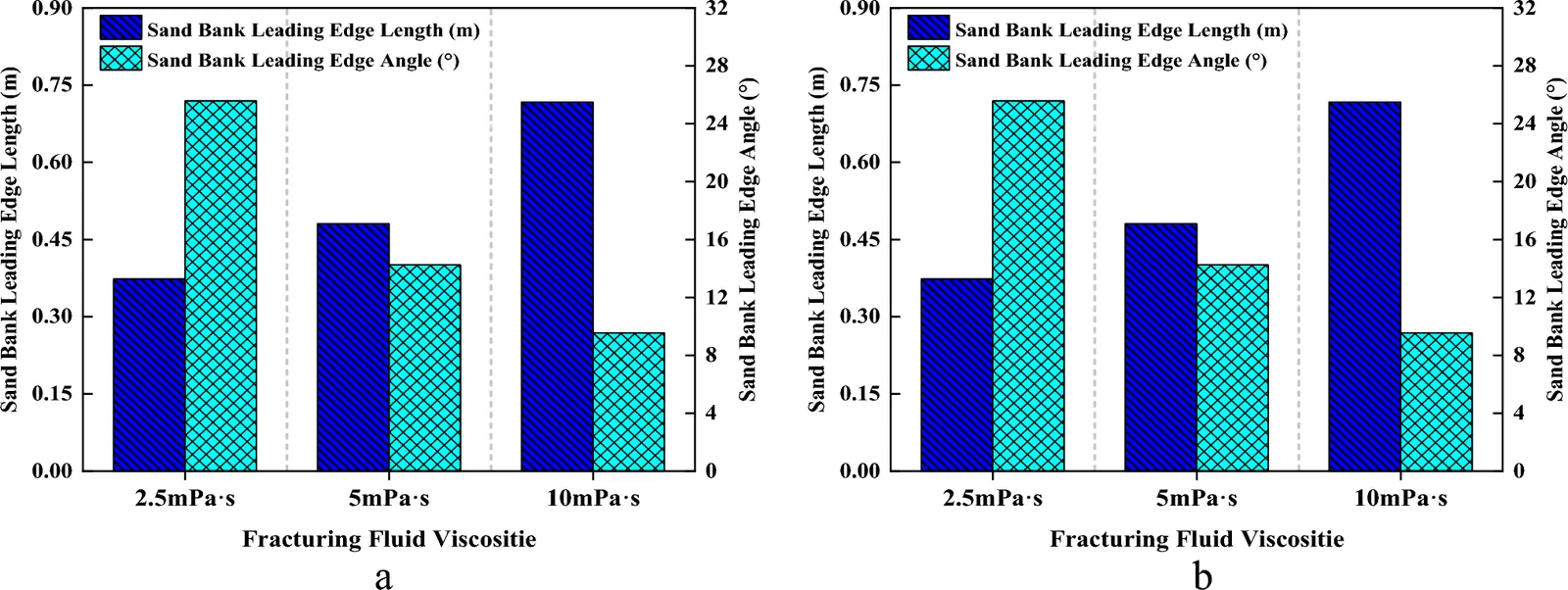
Relationship between characteristic parameters and fracturing fluid viscosity (**a**) Proppant Leading Edge (**b**) Trailing Edge of Proppant.

Comparison of proppant bank morphology under different viscosities shows that as viscosity increases, proppant migrates deeper into the fracture. The proppant bank leading-edge height gradually decreases, while the leading-edge length and main body length increase with viscosity. Compared to variable-width fractures, the constant-width rectangular fracture has significantly lower trailing-edge fill degree. Observing the trailing-edge region in wedge-shaped and elliptical fractures, the elliptical fracture shows higher trailing-edge fill degree, further indicating that width variation in wedge-shaped fractures has a greater impact on proppant transport and placement. As shown in Fig. 9, as fracturing fluid viscosity increases, both the leading-edge length and trailing-edge length of the proppant bank increase, while the inclination angle shows the opposite trend.

**Fig. 10:**
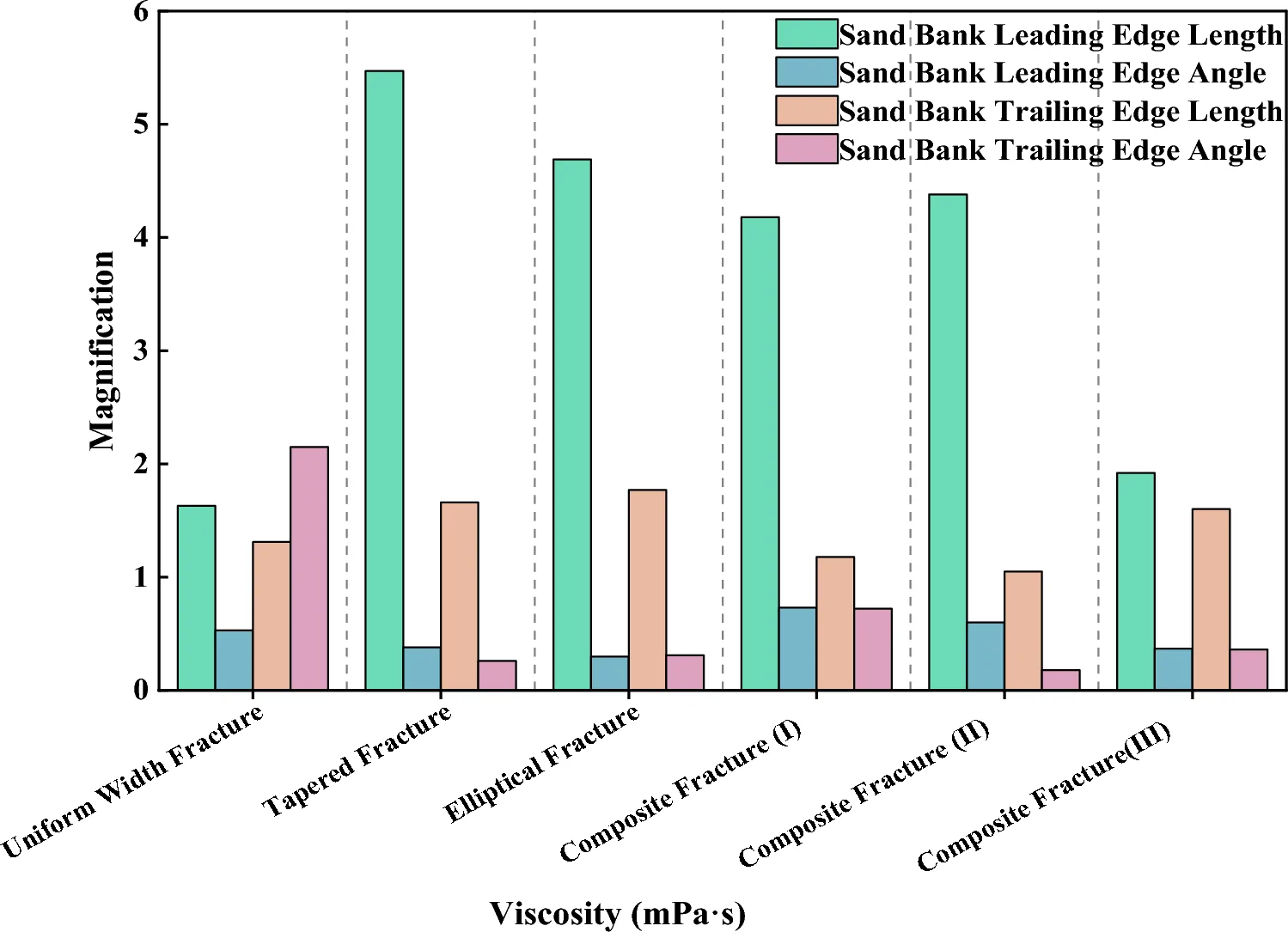
Trend of parameters for different fracture types with fracturing fluid viscosity.

Taking the change from 2.5 mPa·s to 10 mPa·s as the study object. Figure 10 shows the trend of parameters for each fracture type with viscosity. Viscosity significantly impacts proppant bank leading-edge length and trailing-edge inclination angle. The wedge-shaped fracture shows the largest increase in leading-edge length (5.47 times). Composite fracture (II) shows the largest decrease in trailing-edge inclination angle (reducing to 0.18 times the original angle). In contrast to the decreasing trend in trailing-edge angle for variable-width fractures, the constant-width fracture shows an increasing trend (2.15 times increase).

**Fig. 11:**
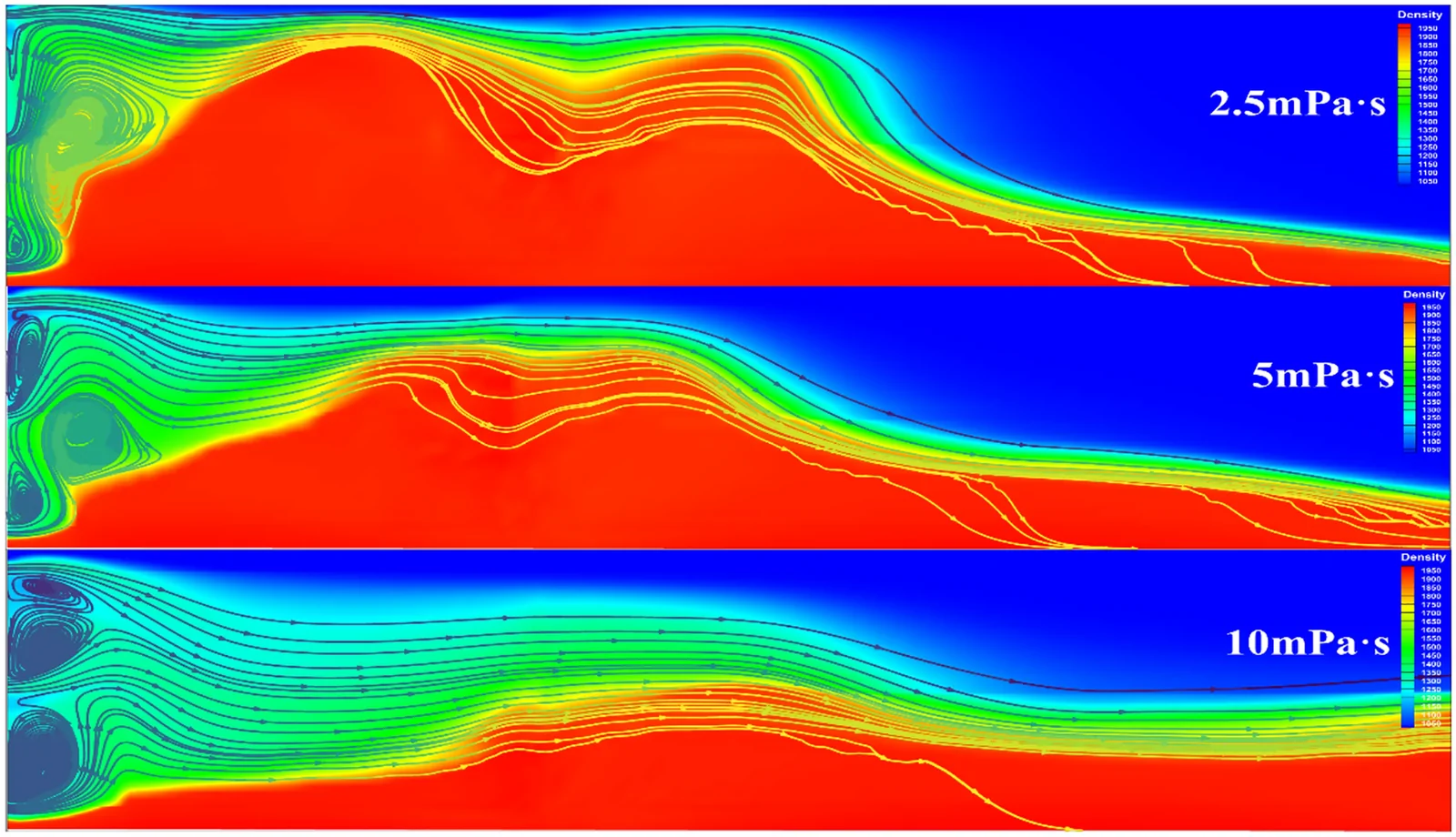
Slurry pathlines within Composite (III) variable-width fracture at different viscosities.

For Composite (III) variable-width fracture at injected sand mass M=9kg and pump time=25s, Fig. 11 shows proppant banks at different viscosities. As viscosity increases, the number of vortices increases from 2 to 3. At low viscosity (2.5 mPa·s), sufficient space near the entrance allows slurry to form one large and one small vortex near inlet 2 and inlet 3, rotating counterclockwise at the bottom and clockwise at the top. At medium viscosity (5 mPa·s), a third vortex forms due to the further extended proppant bank leading-edge. The new vortex rotates counterclockwise, consistent with earlier flow rate observations. At high viscosity (10 mPa·s), high viscosity carries more proppant deeper into the fracture, resulting in an excessively long leading-edge. The counterclockwise vortex formed near inlet 3 fails to interact with the deposited bank, causing only one counterclockwise vortex near inlet 2 and ultimately forcing a clockwise vortex near inlet 1 (a counterclockwise vortex at inlet 1 would cancel with inlet 2’s vortex).

#### Proppant particle size

For the three proppant sizes of 30/50 mesh, 40/70 mesh, and 70/140 mesh, the proppant mass was set to 9 kg, the pumping rate to 18 L/min, and the pumping duration to 75 s. Under the above conditions, the proppant placement morphology in constant-width, wedge-shaped, elliptical, and composite fractures was simulated. Comparison results are shown in Table 11.

**Table 11:**
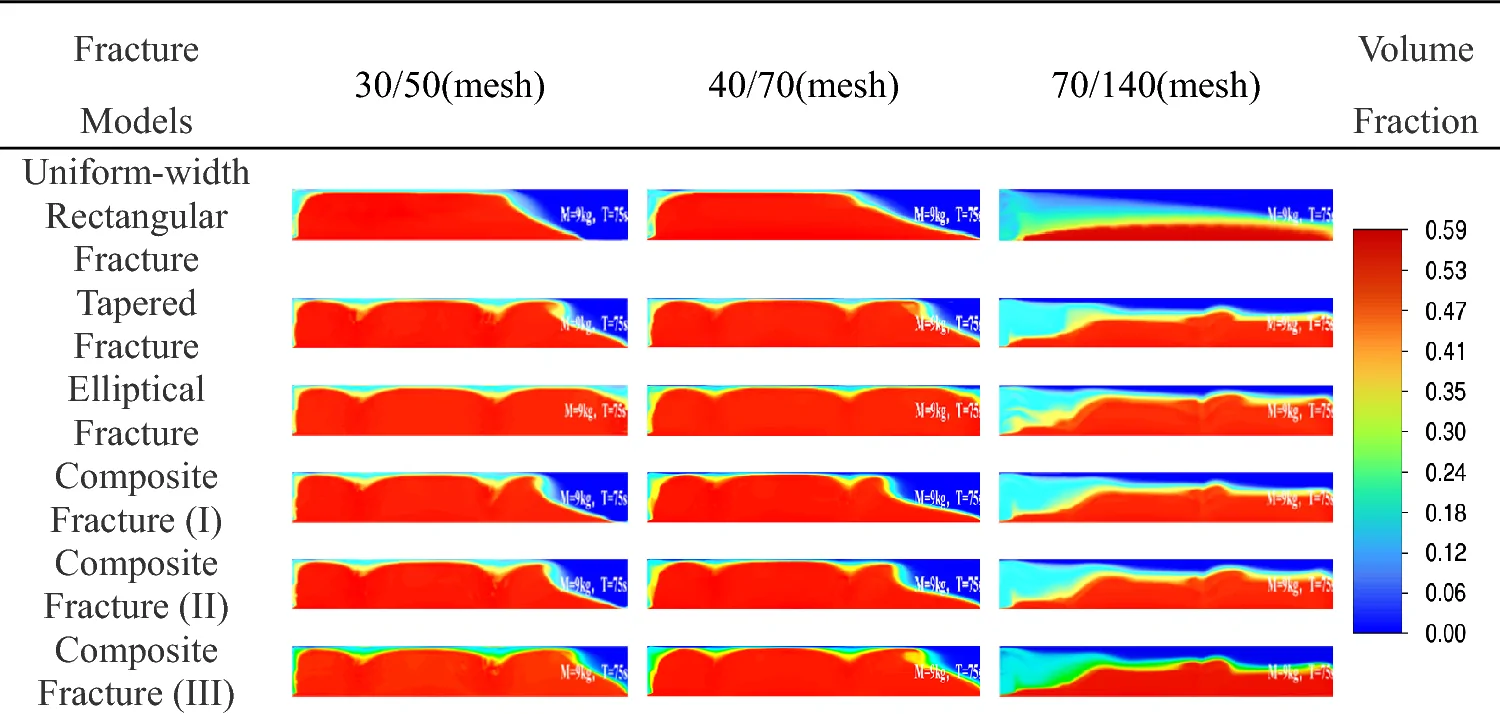
Proppant distribution morphology within fractures for different proppant sizes.

**Fig. 12:**
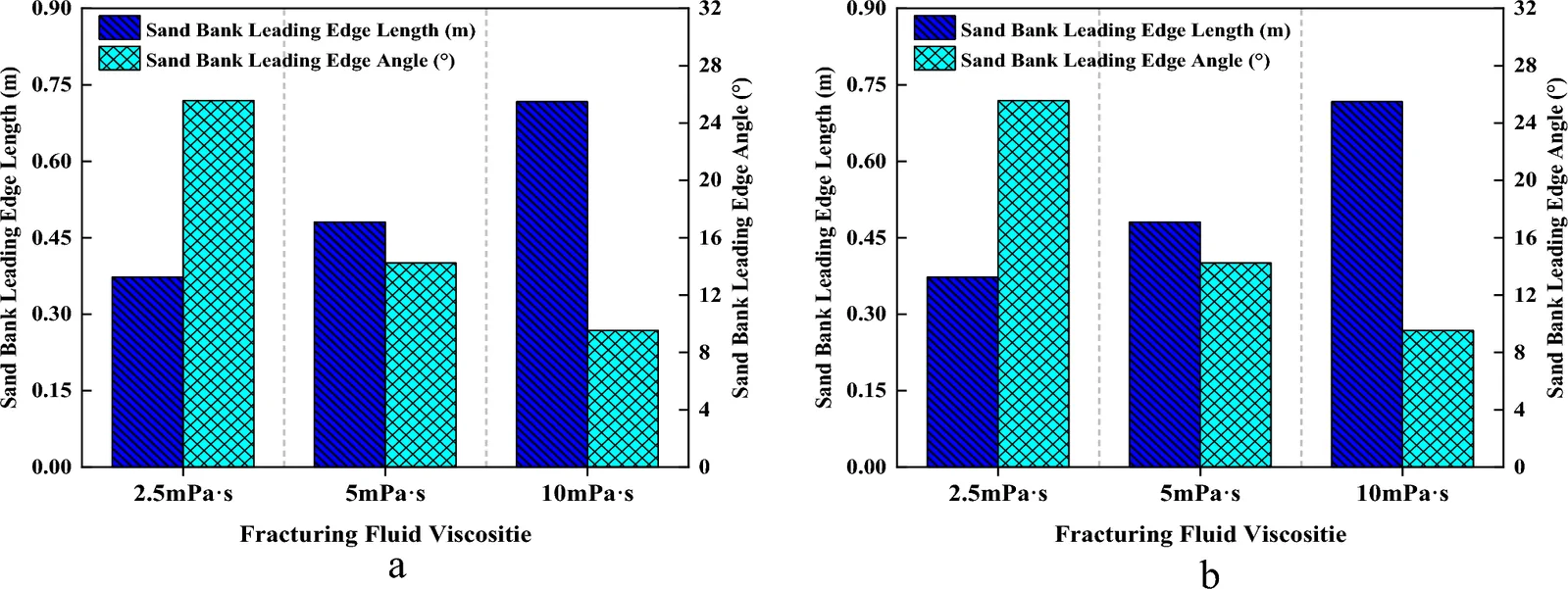
Relationship between characteristic parameters and proppant particle size (**a**) Proppant leading edge (**b**). Trailing edge of proppant.

As proppant particle size decreases, more proppant particles are carried deeper into the fracture by the fracturing fluid. Proppant bank leading-edge length increases, while main body and trailing-edge length decrease. Differences in proppant bank morphology between constant-width and other fracture morphologies are minor for 30/50 and 40/70 mesh proppant. However, with the smaller 70/140 mesh proppant, significant differences arise, the most prominent being a significantly lower equilibrium height in the constant-width fracture. Comparing the trailing-edge region in wedge-shaped and elliptical fractures, the elliptical fracture shows higher trailing-edge fill degree, further indicating wedge-shaped width variations have a greater impact on proppant transport and placement. Figure 12 shows that as proppant particle size decreases, both leading-edge and trailing-edge lengths gradually increase, with the rate of increase accelerating. The trailing-edge inclination angle also increases, while the leading-edge inclination angle shows the opposite trend.

**Fig. 13:**
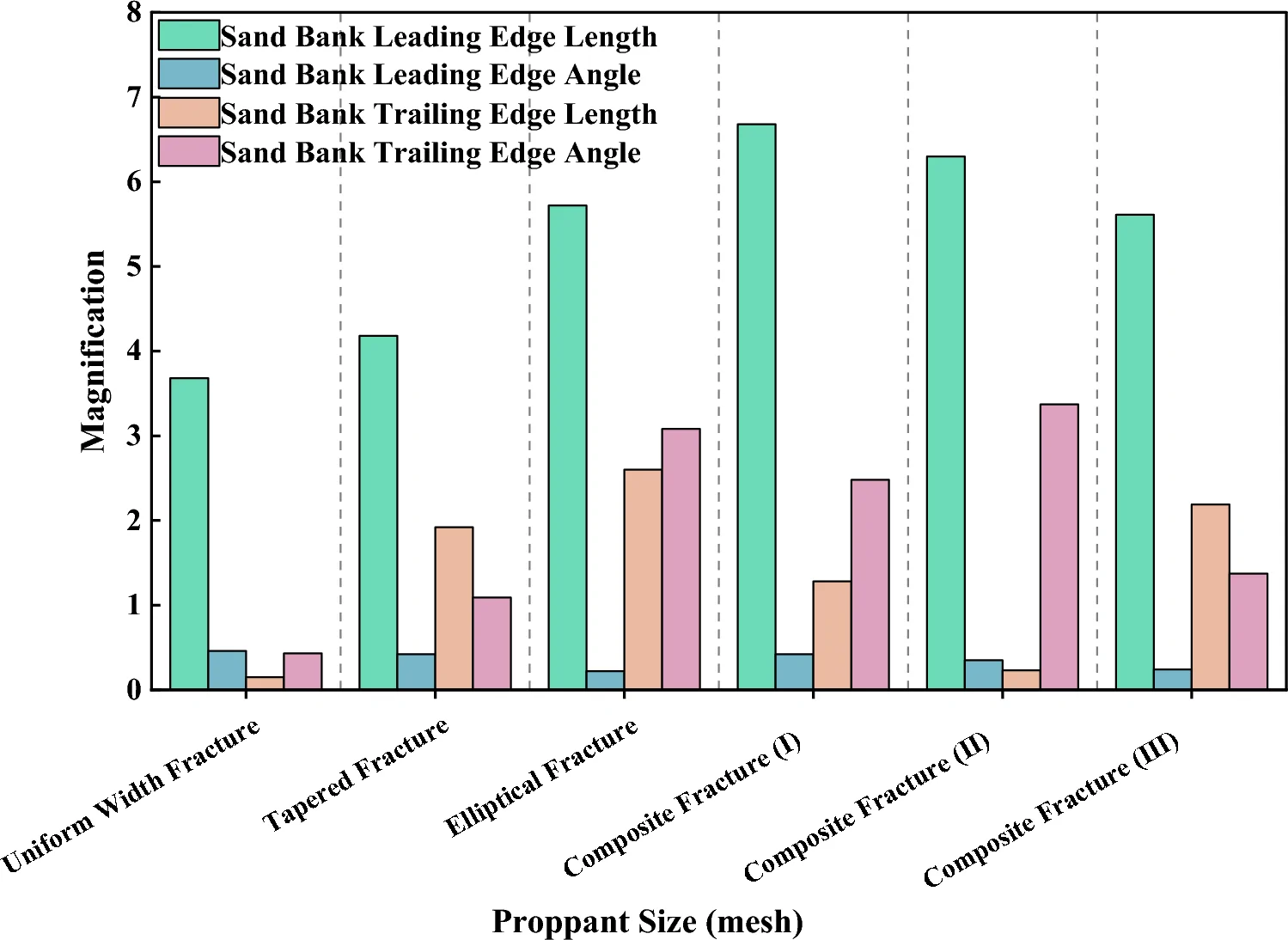
Trend of parameters for different fracture types with proppant particle size.

Taking the change from 30/50 mesh to 70/140 mesh as the study object. Figure 13 shows the trend of parameters for each fracture type with particle size. Particle size significantly impacts proppant bank leading-edge length, trailing-edge length, and trailing-edge inclination angle. Composite fracture (I) shows the largest increase in leading-edge length (6.68 times). The constant-width fracture shows the largest decrease in trailing-edge length (0.15 times the original), while Composite fracture (II) shows the largest increase in trailing-edge length (3.37 times). Except for the constant-width fracture, where the trailing-edge angle decreases, other variable-width fractures show an increase in trailing-edge angle.

**Fig. 14:**
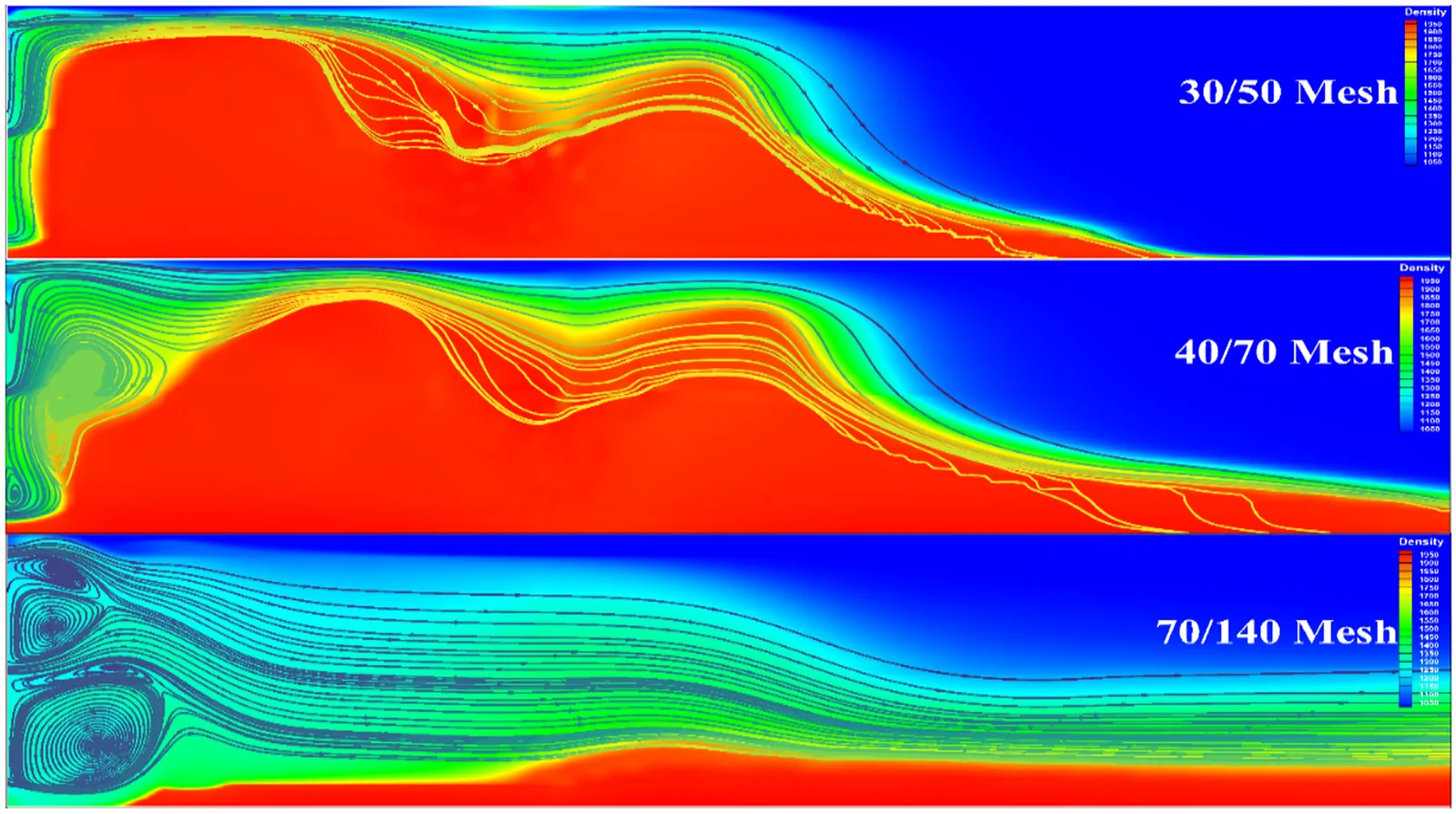
Slurry pathlines within composite (III) variable-width fracture for different proppant sizes.

For Composite (III) variable-width fracture at injected sand mass M=9kg and pump time=25s, Fig. 14 shows slurry pathlines for different particle sizes. As particle size decreases, the number of vortices increases. At large particle size (30/50 mesh), excessive accumulation near the inlet creates very narrow space, preventing vortex formation. At medium particle size (40/70 mesh), sufficient space exists for vortex formation. Slurry forms a counterclockwise vortex near inlet 1; encountering the proppant bank, it merges with slurry from inlet 2 to form a larger clockwise vortex. At small particle size (70/140 mesh), proppant is easily carried far into the fracture, leaving a large unfilled region near the entrance. Due to the long leading-edge, the counterclockwise vortex near inlet 1 cannot interact with the bank to maintain its rotation, so only one counterclockwise vortex forms near inlet 2, forcing a clockwise vortex near inlet 1.

#### Proppant concentration (sand ratio)

For the three sand ratios of 10%, 15%, and 20%, the proppant mass was set to 9 kg, the pumping rate to 18 L/min, and the pumping duration to 75 s. Under the above conditions, the proppant placement morphology in constant-width, wedge-shaped, elliptical, and composite fractures was simulated. Comparison results are shown in Table 12.

**Table 12:**
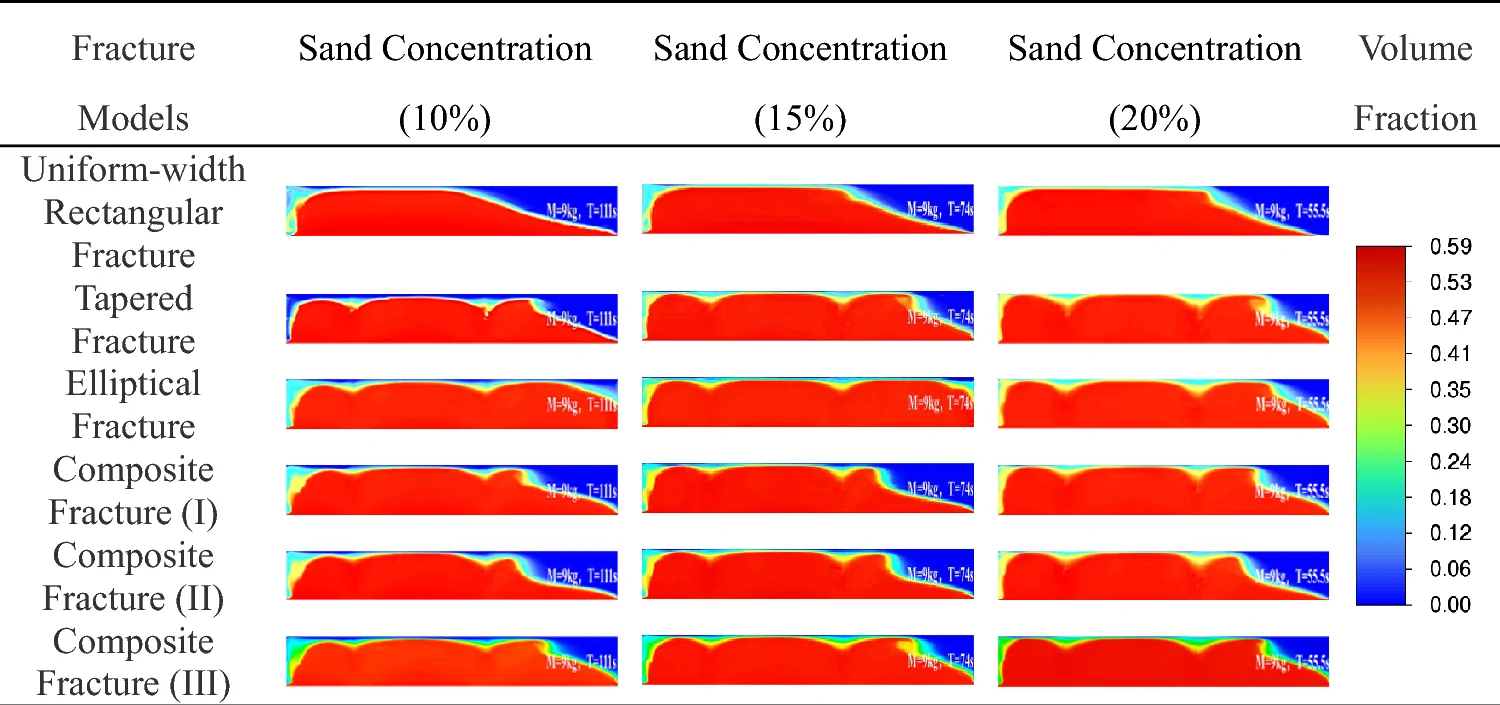
Proppant distribution morphology within fractures at different sand ratios.

**Fig. 15:**
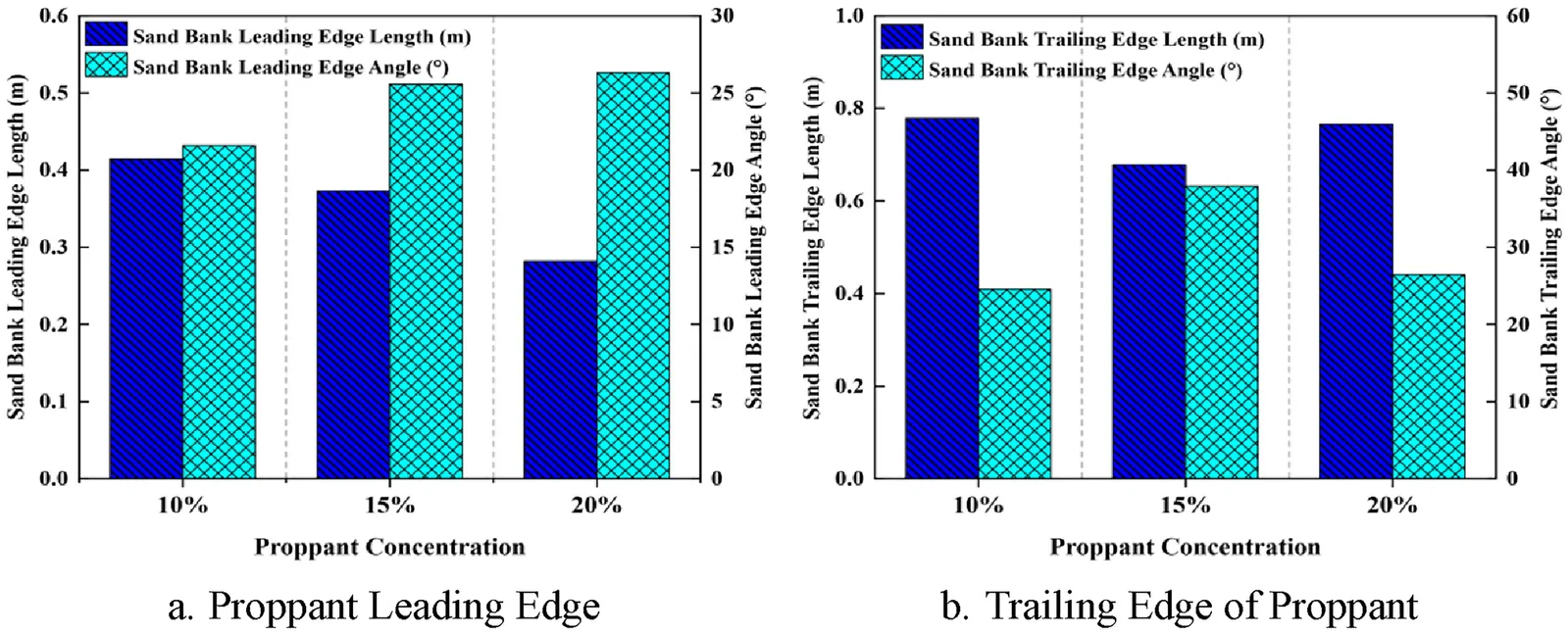
Relationship between characteristic parameters and sand ratio.

As proppant concentration increases, the overall placement length and leading-edge length of the proppant bank decrease. This is mainly due to increased proppant concentration at high sand ratios, leading to greater transport resistance and significantly enhanced local accumulation effects, which inhibit leading-edge extension and promote proppant bank height elevation. Comparing different fracture morphologies, the constant-width rectangular fracture has the lowest fill degree. Among variable-width fractures, the elliptical fracture shows higher fill degree in the trailing-edge region. Figure 15 shows that as sand ratio increases, proppant bank leading-edge length gradually decreases, leading-edge inclination angle gradually increases, trailing-edge length first decreases then increases, and trailing-edge inclination angle shows the opposite trend.

**Fig. 16:**
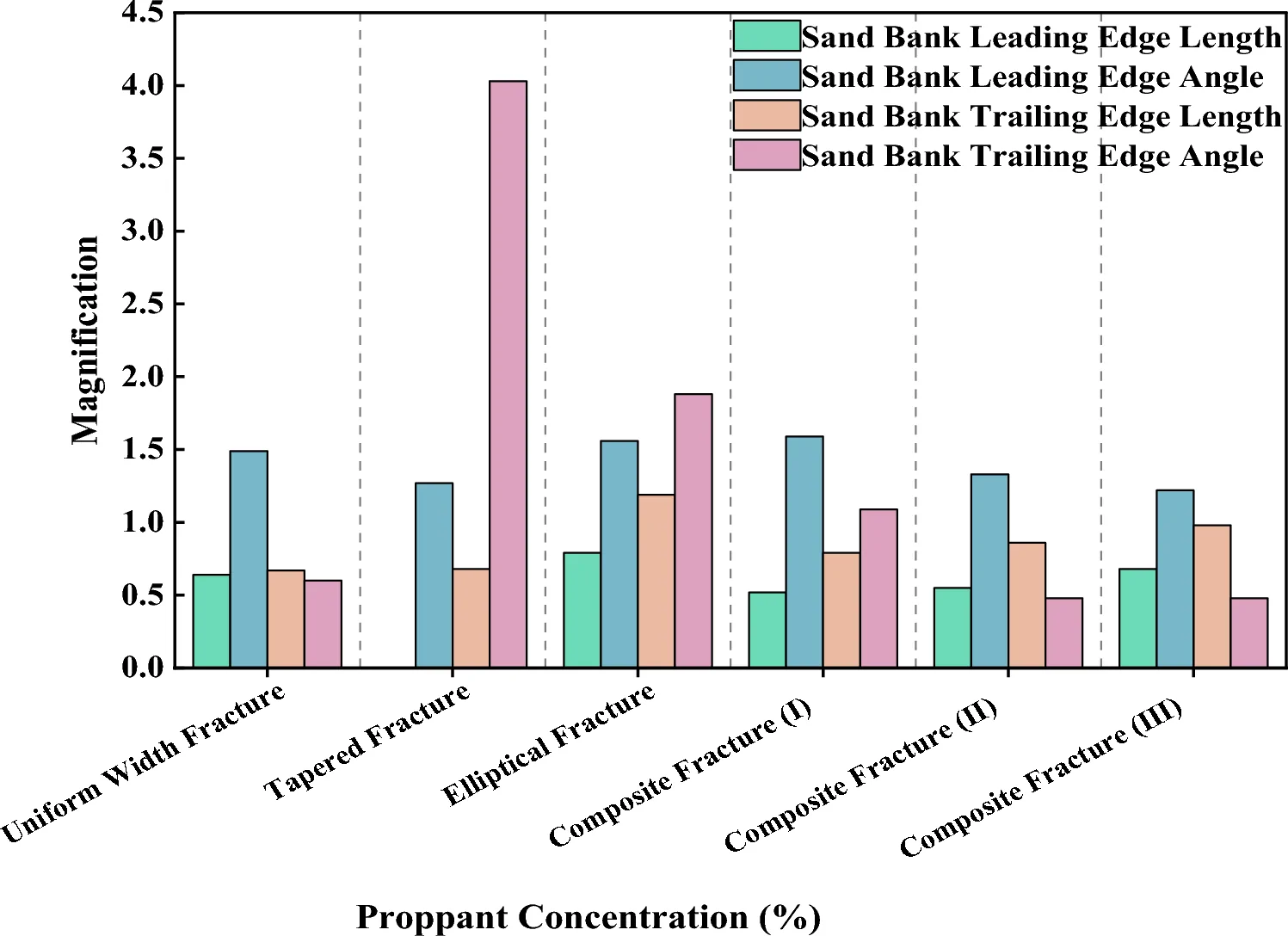
Trend of parameters for different fracture types with proppant concentration.

Taking the change from 10% to 20% sand ratio as the study object. Figure 16 shows the trend of parameters for each fracture type with sand ratio. Sand ratio significantly impacts proppant bank leading-edge inclination angle and trailing-edge inclination angle. Composite fracture (I) shows the largest increase in leading-edge length (1.59 times). The wedge-shaped fracture shows the largest increase in trailing-edge inclination angle (4.03 times). Changes in leading-edge inclination angle and trailing-edge length with sand ratio are relatively moderate compared to other factors.

**Fig. 17:**
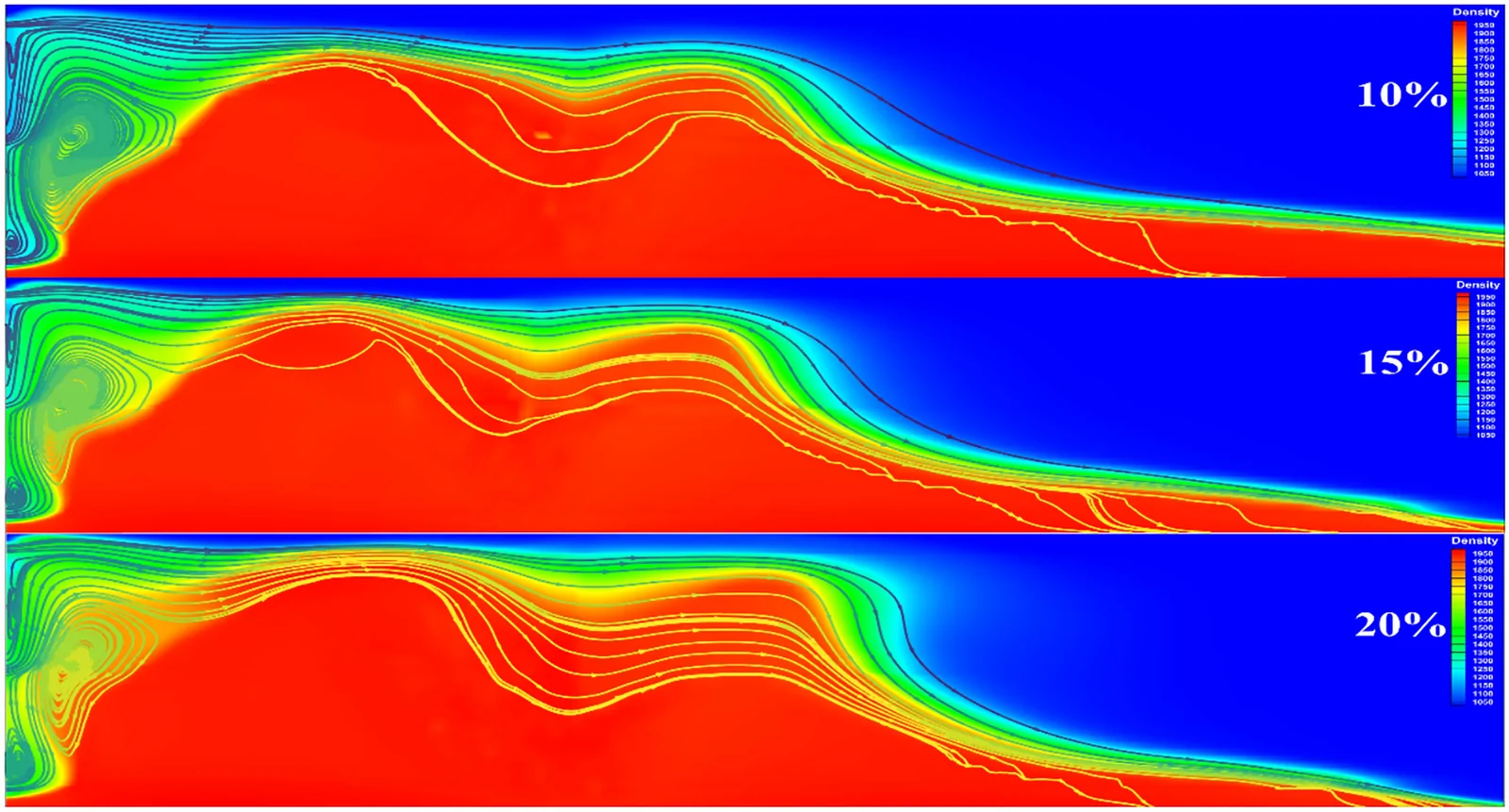
Slurry pathlines within composite (III) variable-width fracture at different sand ratios.

For Composite (III) variable-width fracture at injected sand mass M=9kg and pump time=25s, Fig. 17 shows slurry pathlines at different sand ratios. As sand ratio increases, a larger clockwise vortex forms near inlet 2 interacting with the bank at all sand ratios, while counterclockwise vortices near inlet 1 and inlet 3 gradually enlarge. The vortex and proppant bank morphologies are relatively similar under all three sand ratios, indicating the influence of sand ratio is relatively minor.

#### Recommendations for fracture morphology-specific parameters

In field practice, fracture geometries can be inferred from microseismic monitoring, tracer tests, or other diagnostic methods. Based on the identified fracture morphology, differentiated recommendations for operational parameters are provided as follows.

##### Rectangular constant-width fracture

A moderate pumping rate, low-to-medium viscosity fracturing fluid, medium proppant size, and a moderate sand ratio are recommended. Under this combination, the front and rear edge lengths of the proppant bank are appropriate, the ineffective supporting area is small, and the proppant placement pattern is well-balanced. An excessively low pumping rate leads to near-wellbore accumulation, while an overly high rate results in excessive front-edge extension and insufficient rear-edge support. Low-to-medium viscosity avoids both weak transport at low viscosity and abnormal increase in rear-edge dip angle at high viscosity. Medium proppant size offers the best consistency across fracture types, and a moderate sand ratio balances bank length and bank height.

##### Wedge-shaped fracture

A high pumping rate, relatively high viscosity, medium proppant size, and moderate sand ratio are recommended. The high pumping rate helps proppant overcome flow resistance in the narrow section and migrate farther, reducing near-wellbore accumulation. Higher viscosity most significantly increases the front-edge length, facilitating transport through the variable-width region. Medium proppant size prevents clogging by large particles and the decline in rear-edge filling by small particles. A moderate sand ratio avoids drastic changes in the rear-edge dip angle.

##### Elliptical fracture

A high pumping rate, low-to-medium viscosity, medium proppant size, and moderate sand ratio are recommended. The elliptical fracture exhibits the shortest rear-edge length and the farthest proppant transport distance; a high pumping rate further enhances this advantage. Low-to-medium viscosity ensures a balanced front and rear edge, preventing excessive front-edge length caused by high viscosity. Medium proppant size achieves a relatively high degree of rear-edge filling under all particle size conditions. The effect of sand ratio is relatively mild, making a moderate sand ratio a general recommendation.

##### Composite fracture Type I

A moderate pumping rate, low-to-medium viscosity, medium proppant size, and moderate sand ratio are recommended. At high pumping rates, the rear-edge dip angle reduction is largest, which is detrimental to rear-edge support. Low-to-medium viscosity avoids limited vortex formation at low viscosity and excessive front-edge length at high viscosity. This fracture type is most sensitive to proppant size: small particles cause an excessively large increase in front-edge length, so medium size is chosen to control the front edge. A moderate sand ratio balances changes in the front-edge dip angle.

##### Composite fracture Type II

A moderate pumping rate, low-to-medium viscosity, small proppant size, and moderate sand ratio are recommended. A moderate pumping rate maintains rear-edge stability. Low-to-medium viscosity prevents an excessive decrease in rear-edge dip angle (this fracture type shows the largest reduction in rear-edge dip angle with viscosity). As proppant size decreases, the rear-edge length increases most significantly for this fracture type; therefore, small proppant size is recommended to fully exploit its variable-width characteristics. A moderate sand ratio is a safe choice.

##### Composite fracture Type III

A moderate pumping rate, low-to-medium viscosity, medium proppant size, and moderate sand ratio are recommended. Under moderate pumping rate, the interaction between vortices and the proppant bank is most harmonious, while a high rate tends to alter vortex directions and cause interference among injection ports. Low-to-medium viscosity promotes multiple vortices, resulting in the most uniform proppant placement. Large proppant size yields no vortices, while small size leads to excessive front-edge length and reversed vortices; medium size produces the most reasonable vortex pattern. Sand ratio has the least influence, with similar vortex patterns observed at different sand ratios, so a moderate sand ratio ensures stable transport.

### Proppant transport laws in fracture networks

#### Fracture network morphology

**Fig. 18:**
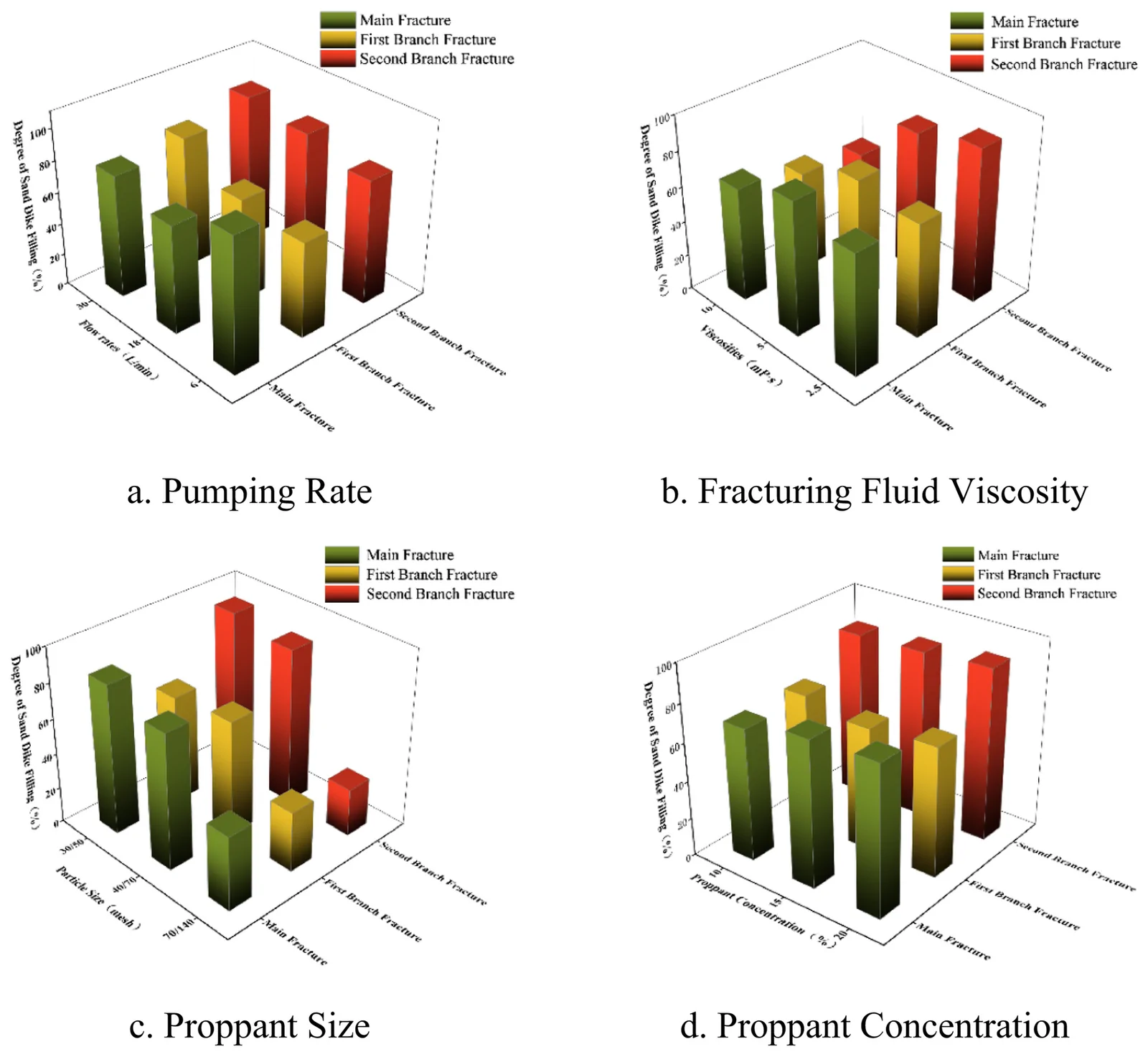
Proppant bank fill degree within the fracture network under different parameter conditionsa. Pumping Rate b. Fracturing Fluid Viscosity c. Proppant Size d. Proppant Concentration.

The placement morphology of the proppant bank within the fracture network is shown in Table 13. Combined with analysis from Fig. 18, as pump rate increases, the leading-edge length of the main fracture proppant bank gradually increases, while the trailing-edge length gradually decreases. The fill degree within primary and secondary branch fractures increases continuously.

**Table 13:**
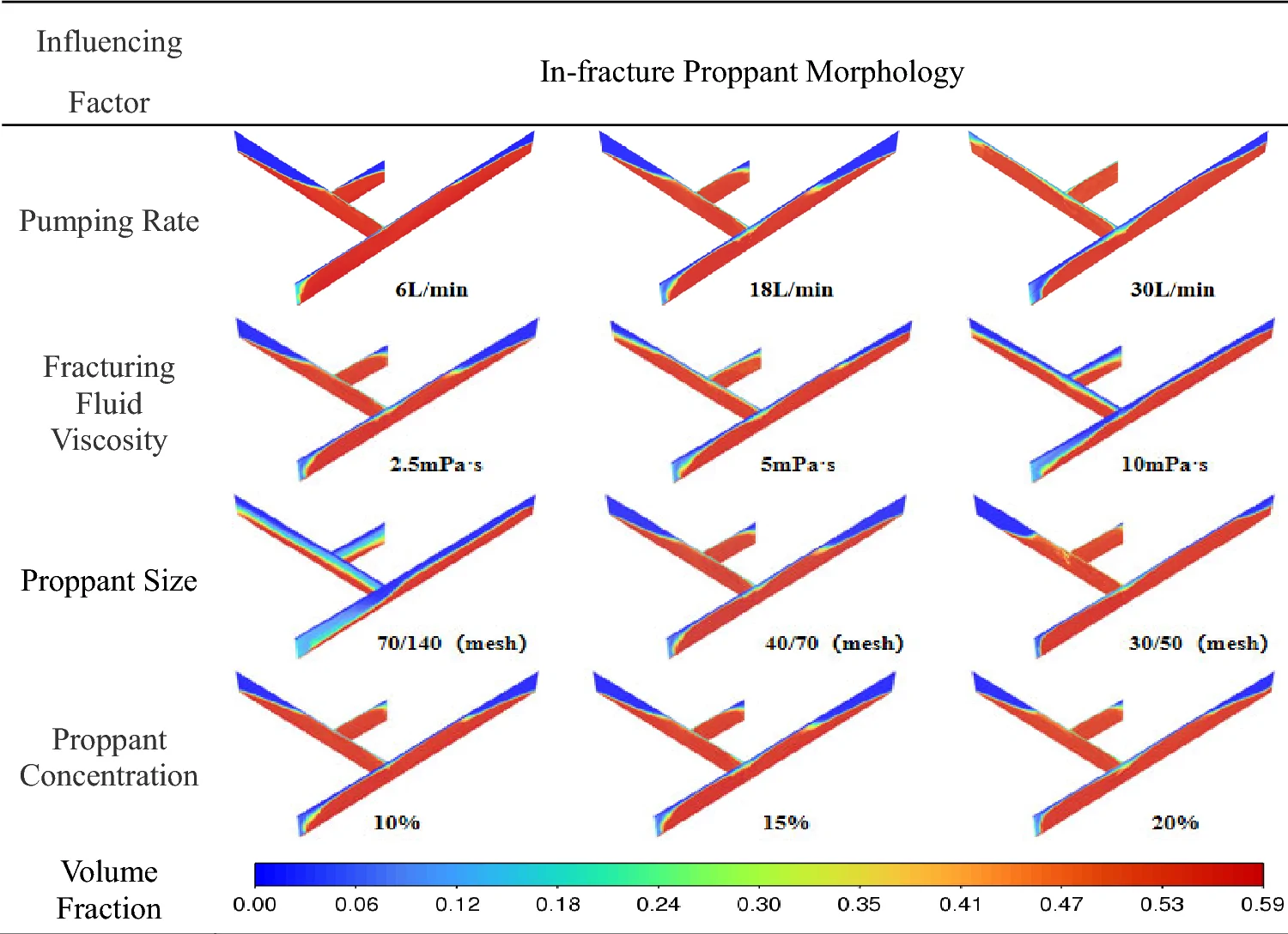
Proppant distribution morphology within the fracture network under different influencing factors.

As viscosity increases, the fill degree of the main body within the main fracture gradually decreases, while the trailing-edge fill degree gradually increases. At viscosities of 2.5 mPa·s and 5 mPa·s, the primary and secondary branch fractures follow the same trend as the main fracture. However, at 10 mPa·s viscosity, the fill degree in primary and secondary branch fractures decreases. This is because high-viscosity fracturing fluid has stronger proppant carrying capacity, transporting more proppant to the far end of the fracture, reducing the amount entering the branch fractures.

As proppant particle size increases, the fill degree near the leading-edge and main body increases. Trailing-edge length first increases then decreases with particle size. At small particle sizes, more proppant is transported far into the fracture, less enters the branch fractures, resulting in lower fill degree. At large particle sizes, limited carrying capacity causes more proppant to accumulate near the fracture front, resulting in lower branch fracture fill degree compared to medium particle sizes.

As sand ratio increases, the leading-edge fill degree in the main fracture gradually increases, trailing-edge length first increases then decreases, and trailing-edge length within branch fractures first decreases then increases.

#### Pump rate

**Fig. 19:**
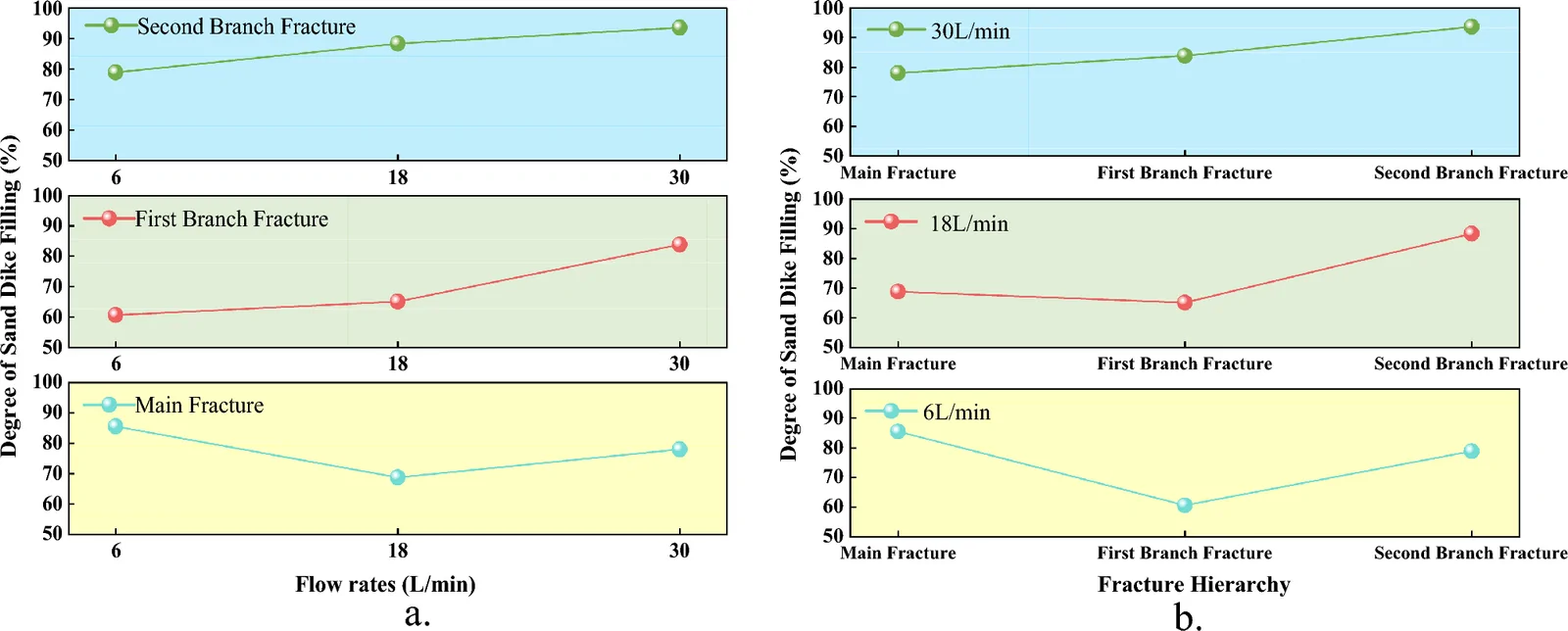
Relationship curves between proppant bank fill degree and pump rate (**a**) Proppant bank filling degree versus pumping rate relationship curve (**b**) Proppant bank filling degree versus fracture stage relationship curve.

By comparing proppant distribution morphology within the main fracture and branch fractures at different flow rates (Table 14), at low flow rate (6 L/min), proppant tends to settle and accumulate directly in the near-wellbore region, gradually reaching equilibrium height before being transported backward, eventually achieving overall equilibrium height within the fracture. At medium-high flow rates (18 L/min, 30 L/min), proppant can be transported directly to the middle and rear sections of the fracture, promoting rapid attainment of equilibrium height for the entire bank. Proppant bank accumulation patterns in branch fractures are similar to the main fracture. Figure 19a shows that as flow rate increases, fill degree within primary and secondary branch fractures continuously increases, while main fracture fill degree first decreases then increases. Figure 19b shows that at low (6 L/min) and medium (18 L/min) flow rates, fill degree decreases from main fracture to secondary branch fracture then increases, while at high flow rate (30 L/min), fill degree increases continuously with fracture level.

**Table 14:**
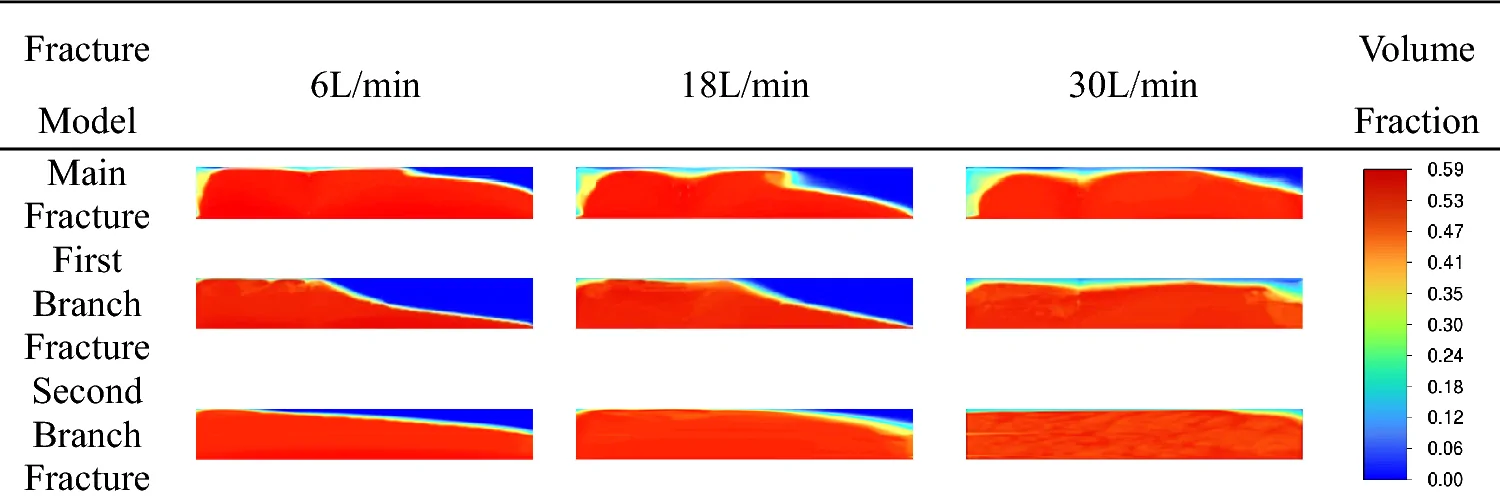
Proppant distribution morphology in main and branch fractures at different pump rates.

**Fig. 20:**
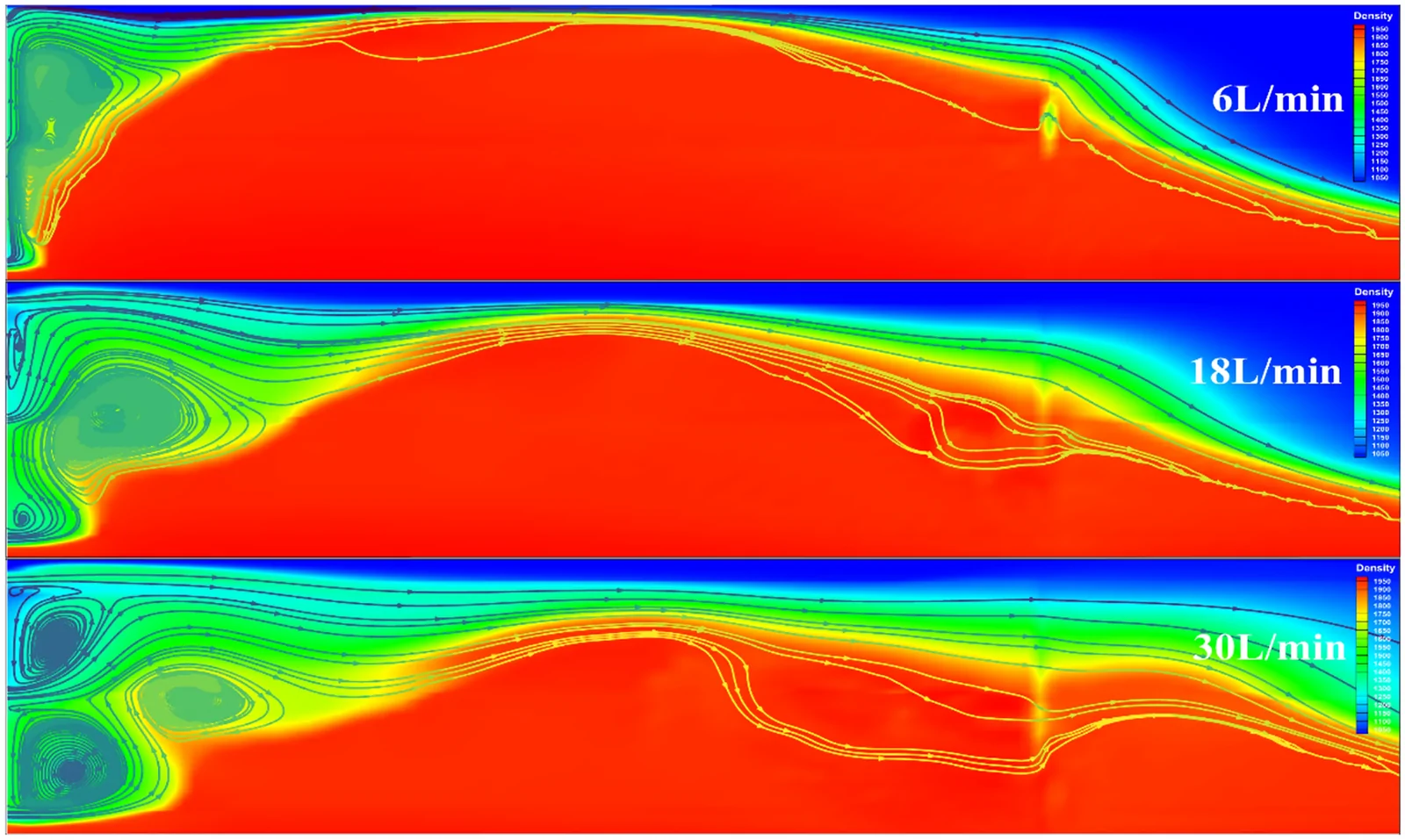
Slurry pathlines within the main fracture at different pump rates.

For the main fracture of the fracture network at injected sand mass M=9kg and pump time=25s, Fig. 20 shows slurry pathlines at different flow rates. As flow rate changes, the vortex morphology changes significantly at 18 L/min, where one clockwise vortex forms. At 6 L/min and 30 L/min, one clockwise and two counterclockwise vortices form.

#### Fracturing fluid viscosity

**Fig. 21:**
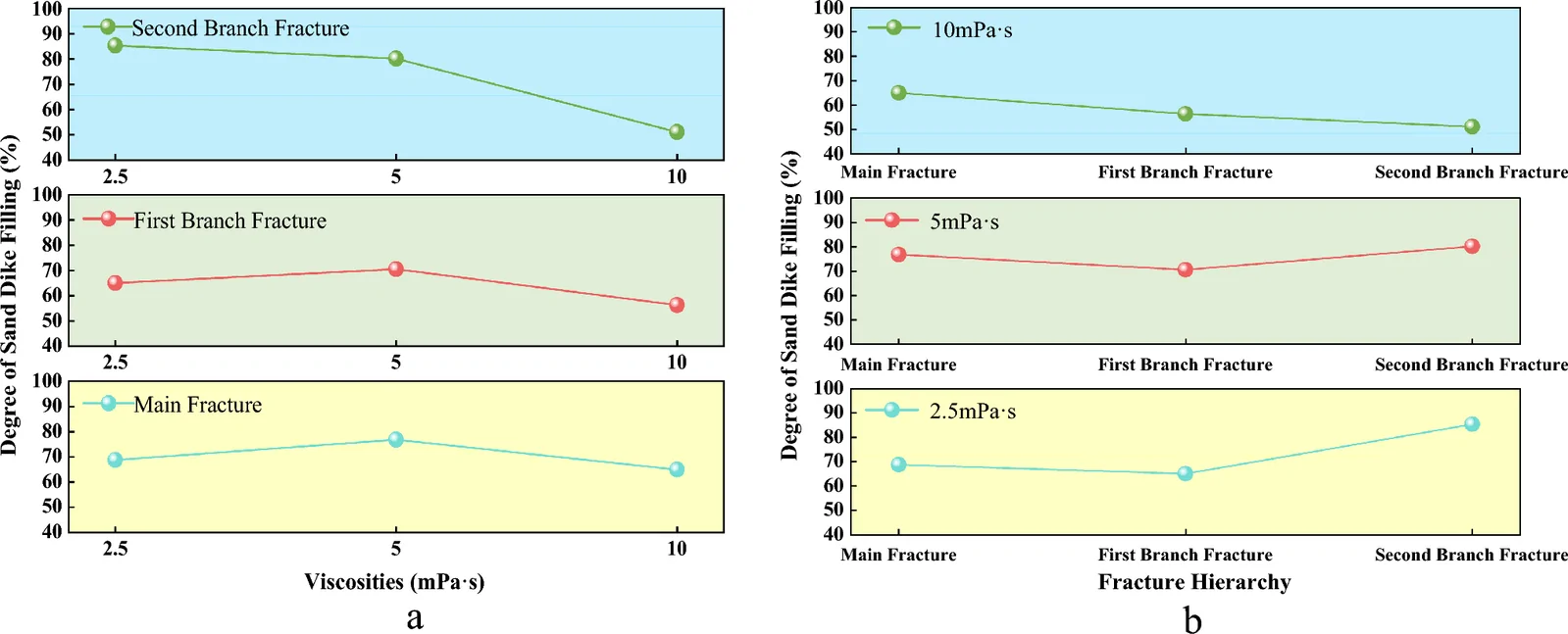
Relationship curves between proppant bank fill degree and viscosity.

Comparing proppant distribution morphology in main and branch fractures at different viscosities (Table 15), as viscosity increases, proppant in the main fracture and primary branches migrates further towards the fracture tip. However, in secondary branches, proppant fill degree first increases then decreases with viscosity. At low viscosity, most proppant accumulates near the wellbore, relatively less enters the branches, hence medium viscosity increases branch fill degree. At high viscosity, more proppant is carried far into the main fracture, reducing the amount entering branches and decreasing branch fill degree. Combined with fill degree analysis for the same fracture level and viscosity (Fig. 21), Fig. 21a shows that as viscosity increases, main fracture and primary branch fill degree first increase then decrease, while secondary branch fill degree gradually decreases with increasing magnitude. Figure 21b shows that at medium (5 mPa·s) and low (2.5 mPa·s) viscosities, the trend of fill degree from main fracture to branches is consistent: slowly decreasing then rapidly increasing. At high viscosity (10 mPa·s), fill degree gradually decreases at a roughly consistent rate.

**Table 15:**
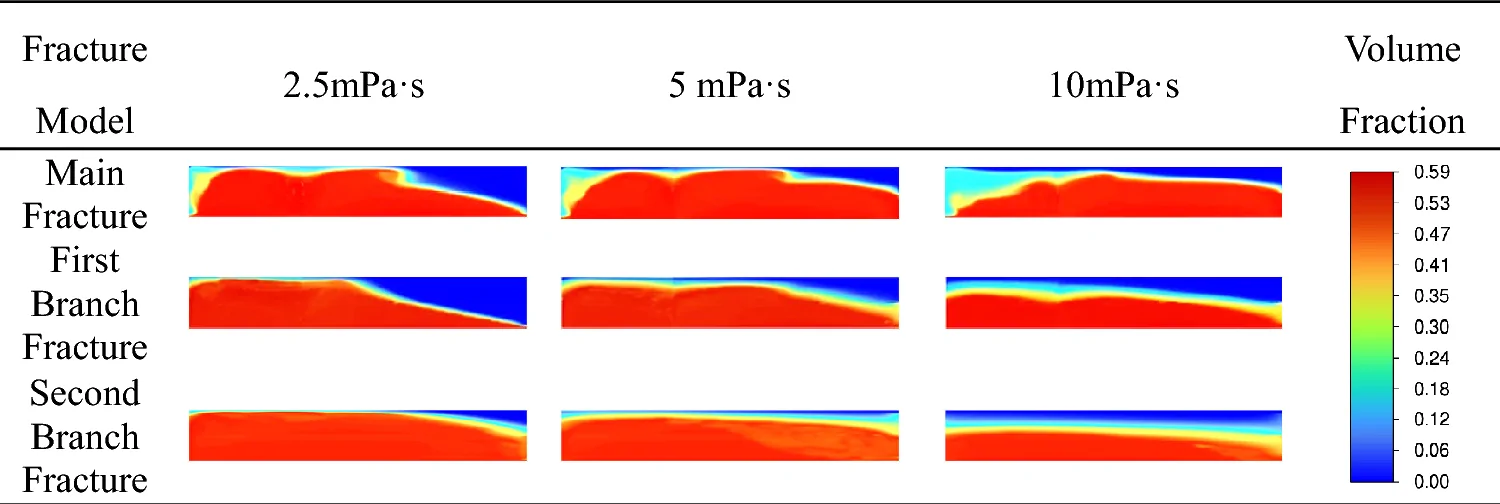
Proppant distribution morphology in main and branch fractures at different fracturing fluid viscosities.

a. Proppant Bank Filling Degree Versus Fracturing Fluid Viscosities Relationship Curve b. Proppant Bank Filling Degree Versus Fracture Stage Relationship Curve

**Fig. 22:**
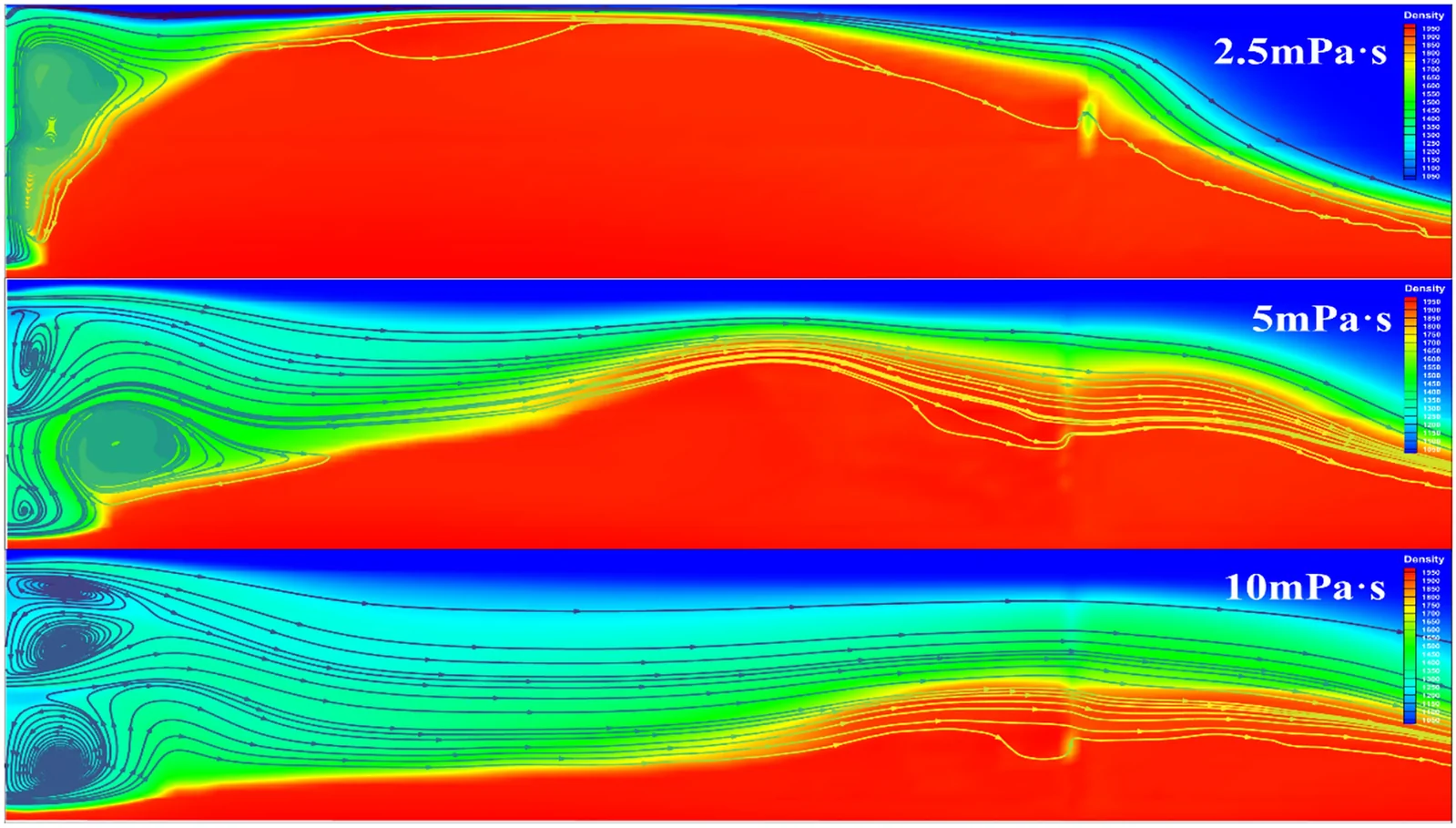
Slurry pathlines within the main fracture at different viscosities.

For the main fracture at pump time=25s, Fig. 22 shows slurry pathlines at different viscosities. The overall vortex situation is similar to the single fracture. At low viscosity (2.5 mPa·s), only one clockwise vortex forms, with minimal vortex effect on the bank; low viscosity also causes excessive accumulation near the leading-edge. At medium viscosity (5 mPa·s), proppant migrates and places further, extending the leading-edge, leading to two counterclockwise vortices and one clockwise vortex formed by interaction with the bank. At high viscosity (10 mPa·s), the vortex near inlet 3 fails to interact with the bank, so one clockwise vortex forms near inlet 1 and two counterclockwise vortices form near inlet 2.

#### Proppant particle size

**Fig. 23:**
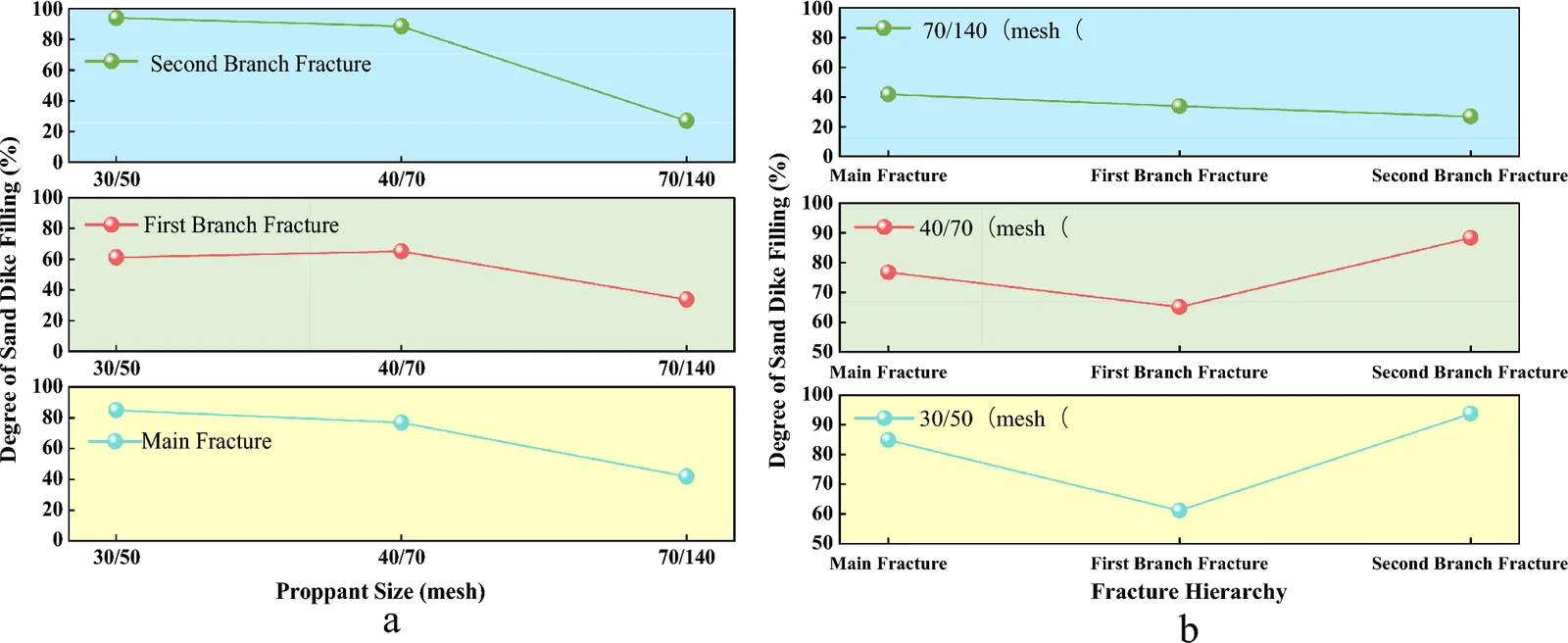
Relationship curves between proppant bank fill degree and proppant size (**a**) Proppant bank filling degree versus proppant size relationship curve (**b**) Proppant bank filling degree versus fracture stage relationship curve.

Comparing proppant placement morphology for different particle sizes (Table 16), combined with fill degree analysis for the same fracture level and particle size (Fig. 23), smaller particle sizes result in proppant placing further into the main fracture, longer leading-edges, and shorter trailing-edges. Most 30/50 mesh proppant accumulates near the wellbore, only migrating further after significant accumulation; hence accumulation is higher near the entrance of primary branches, and more proppant enters secondary branches, resulting in higher fill degree. At 40/70 mesh, more proppant migrates further, and placement within branches follows the main fracture trend. At 70/140 mesh, branch fill degree significantly decreases. This difference arises because smaller, less deposited proppant is mostly transported far into the fracture, with less entering the branches.

**Table 16:**
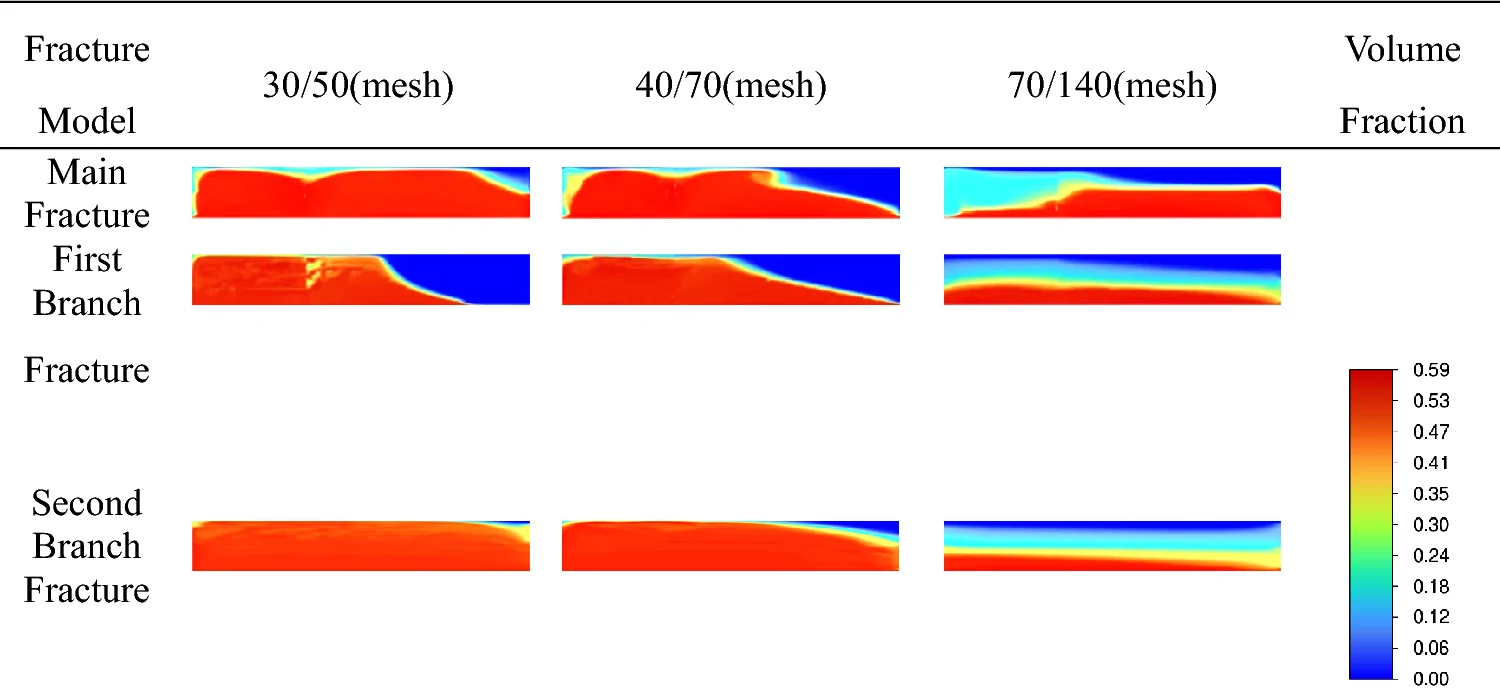
Proppant distribution morphology in main and branch fractures for different proppant sizes.

**Fig. 24:**
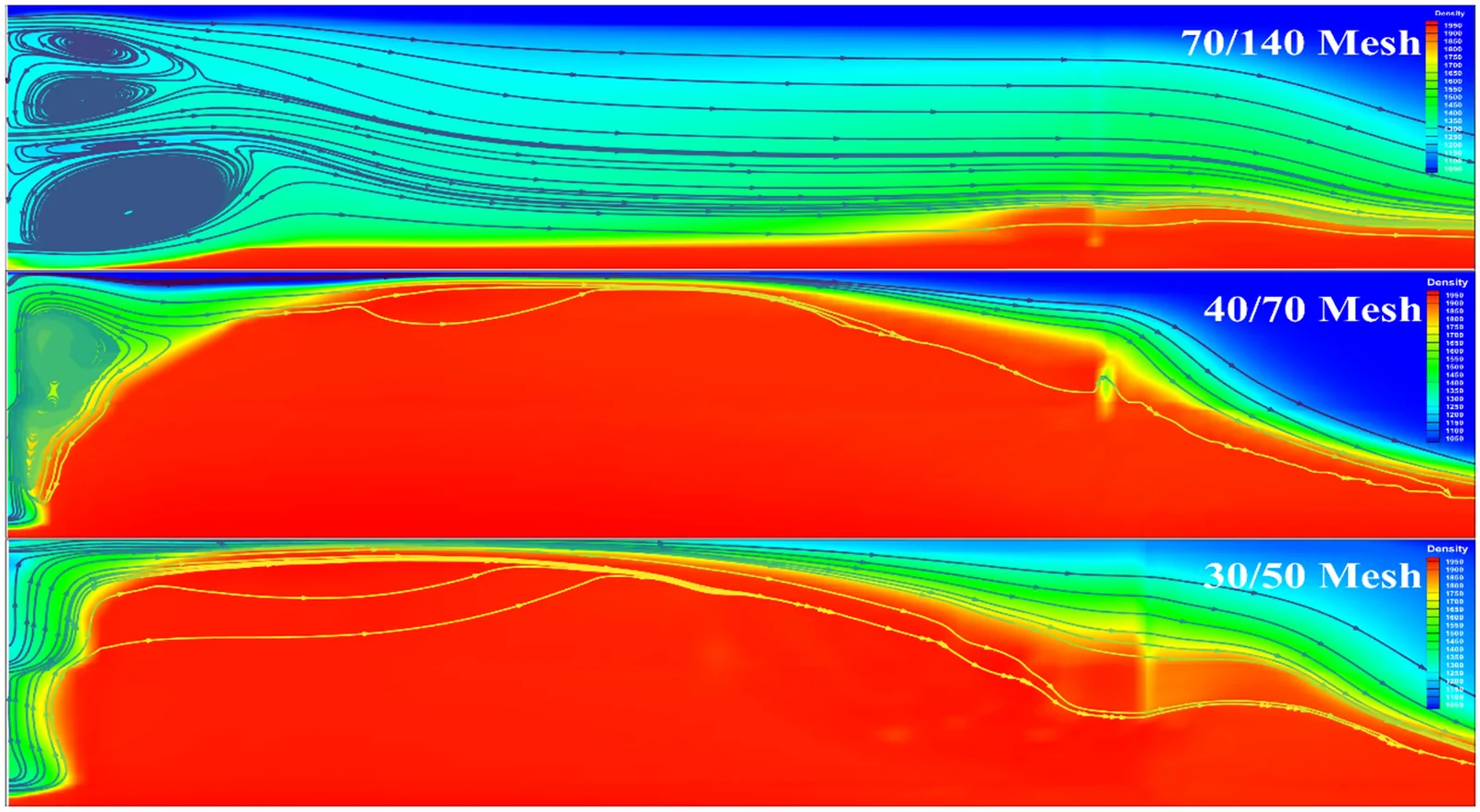
Slurry pathlines within the main fracture for different proppant sizes.

For the main fracture at pump time=25s, Fig. 24 shows slurry pathlines for different particle sizes. As particle size increases, proppant bank fill degree increases. At 70/140 mesh, three vortices form; at 30/50 mesh, no vortices form. Comparing placement morphology for the same size in single fractures, 70/140 mesh proppant places further in the fracture network main fracture. The reason is that some proppant enters the branches, allowing the fracturing fluid to carry the remaining proppant farther.

#### Proppant concentration (sand ratio)

**Fig. 25:**
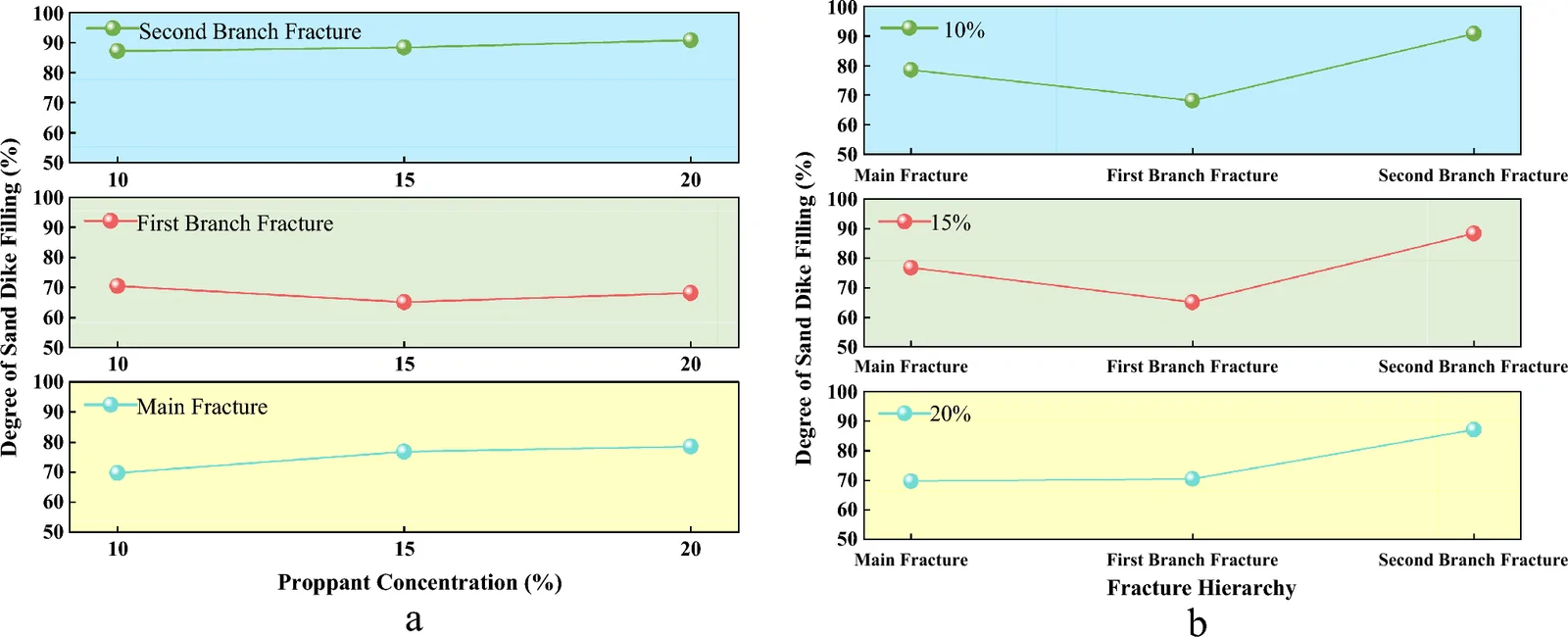
Relationship curves between proppant bank fill degree and sand ratio (**a**) Proppant bank filling degree versus proppant concentration relationship curve (**b**) Proppant bank filling degree versus fracture stage relationship curve.

Comparing placement morphology for different sand ratios (Table 17), combined with fill degree analysis for the same fracture level and sand ratio (Fig. 25), increasing sand ratio significantly affects proppant bank accumulation morphology and characteristics. Higher sand ratio increases proppant deposition efficiency, increasing accumulation near the wellbore, thereby expanding the supported area near the fracture entrance. Leading-edge length decreases, leading-edge inclination angle increases, main body placement length increases, and fill area ratio increases, while trailing-edge length and inclination angle decrease with increasing sand ratio. Higher sand ratio also promotes more uniform proppant distribution in the main fracture body region, extending the main placement length. Main fracture placement area increases with higher sand ratio.

**Table 17:**
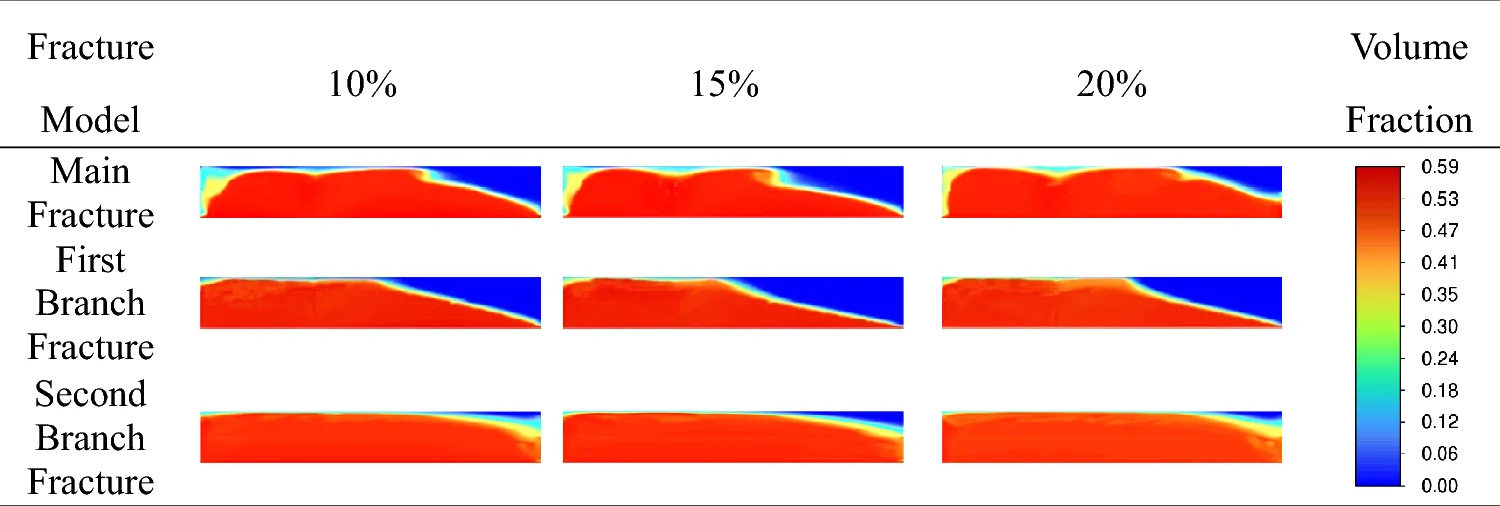
Proppant distribution morphology in main and branch fractures at different proppant concentration.

**Fig. 26:**
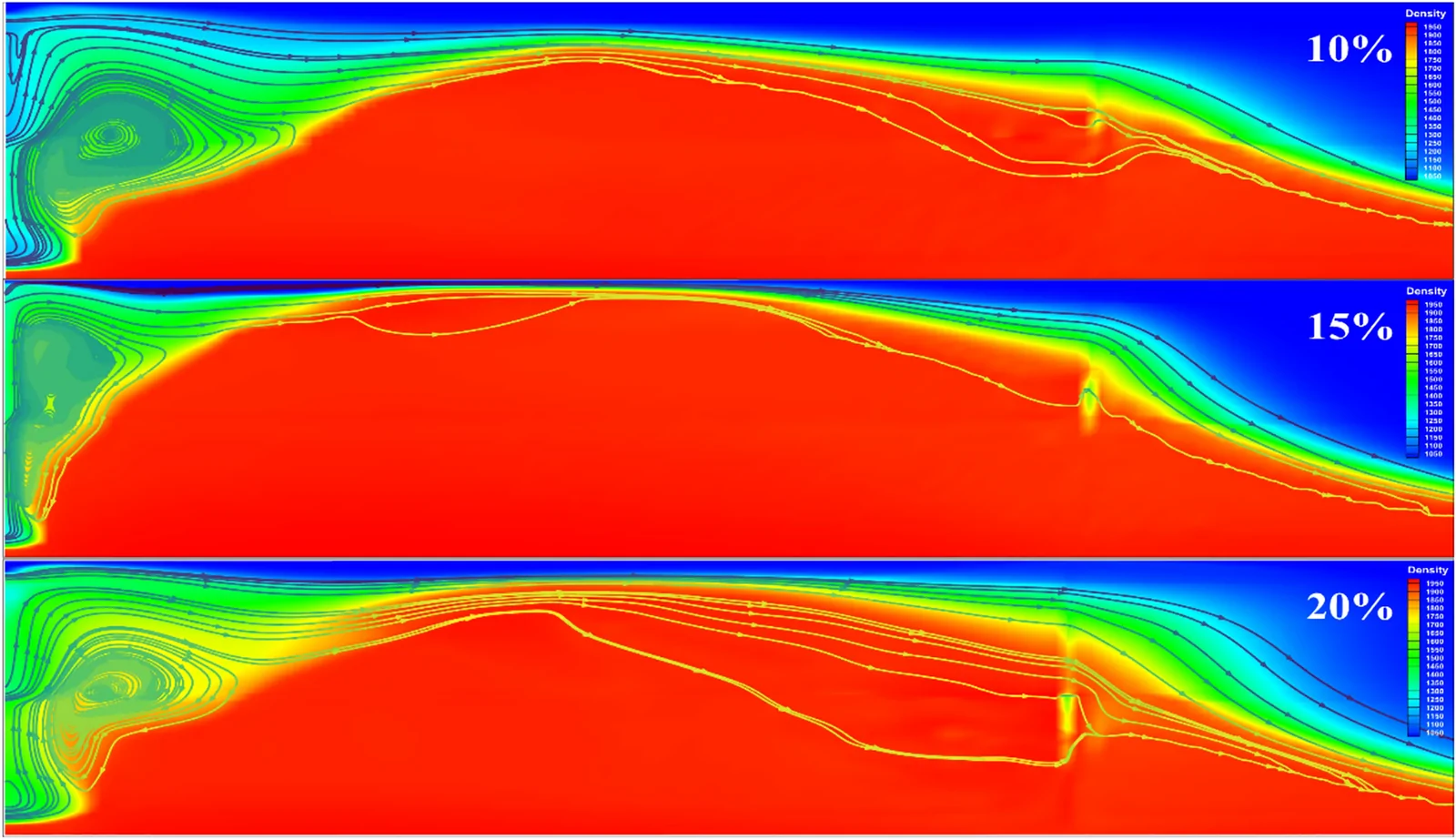
Slurry pathlines within the main fracture at different sand ratios.

For the main fracture at pump time=25s, Fig. 26 shows slurry pathlines at different sand ratios. As sand ratio increases, only one clockwise vortex forms at all ratios; the vortex is smaller at 15% sand ratio, while morphologies at 10% and 20% are relatively similar.

## Conclusion

1. As pump rate increases, the constant-width fracture exhibits the largest increase in proppant bank leading-edge length compared to variable-width fractures, reaching 3.95 times. As viscosity increases, the trailing-edge inclination angle of variable-width fractures shows a decreasing trend, while that of constant-width fractures shows an increasing trend, with an increase magnitude of 2.15 times. As proppant particle size decreases, the trailing-edge inclination angle of constant-width fractures increases, while that of variable-width fractures decreases.
2. In fracture network placement morphology, proppant bank fill degree within fractures at all levels shows a positive correlation with increasing parameters. However, at a proppant size of 70/140 mesh, the leading-edge length in the main fracture significantly increases, resulting in lower fill degree in both main and branch fractures. At a pump rate of 30 L/min, fill degree in primary and secondary branch fractures is significantly higher than under other influencing factors.
3. Comparison of pathlines under various parameters reveals that when space near the fracture entrance is narrow, only one counterclockwise vortex forms near the bottom inlet, conforming to the narrow space. If sufficient space exists between the proppant bank and the fracture entrance, the lower counterclockwise vortex interacts with the bank to form a clockwise vortex slightly away from the middle inlet. Additionally, a slightly smaller clockwise vortex typically forms near the upper inlet. No vortices form in regions far from the fracture entrance.
4. For different fracture morphologies, the recommended operational parameters are as follows: rectangular constant-width fractures – moderate pumping rate, low-to-medium viscosity, medium proppant size, and moderate sand ratio; wedge-shaped fractures – high pumping rate, high viscosity, medium proppant size, and moderate sand ratio; elliptical fractures – high pumping rate, low-to-medium viscosity, medium proppant size, and moderate sand ratio; composite Types I and III – moderate pumping rate, low-to-medium viscosity, medium proppant size, and moderate sand ratio; and composite Type II – moderate pumping rate, low-to-medium viscosity, small proppant size, and moderate sand ratio.

## Data Availability

The data that supports the findings of this study are available within the article.

## References

[1] Wan, S. Evaluation methods of unconventional oil and gas resources and social benefit evaluation. *Autom. Appl.* **64**(06), 7–9 (2023).

[2] Zhu, C. China’s unconventional oil and gas become an important support for increasing reserves and production. *China Pet. Enterp.* **03**, 36–38 (2024).

[3] Stokes G G. On the effect of the internal friction of fluids on the motion of pendulums. (1851).

[4] Kirkby, L. L., Rockefeller, H. A. Proppant settling velocities in nonflowing slurries[C]//SPE Rocky Mountain Petroleum Technology Conference/Low-Permeability Reservoirs Symposium. SPE. SPE-13906-MS. (1985).

[5] Harrington, L. J., Hannah, R. R., Williams, D. Dynamic experiments on proppant settling in crosslinked fracturing fluids[C]//SPE Annual Technical Conference and Exhibition. SPE, 1979: SPE-8342-MS. (1979).

[6] Clark, P. E., Manning, F. S., Quadir, J. A, et al. Prop transport in vertical fractures[C]//SPE Annual Technical Conference and Exhibition. SPE, 1981: SPE-10261-MS. (1981).

[7] Kirkby, L. L., Rockefeller, H. A. Proppant settling velocities in nonflowing slurries[C]//SPE Rocky Mountain Petroleum Technology Conference/Low-Permeability Reservoirs Symposium. SPE, 1985: SPE-13906-MS. (1985).

[8] Cleary, M. P., Fonseca, Jr A. Proppant Convection and Encapsulation in Hydraulic Fracturing; Practical Implications of Computer and Laboratory Simulations[C]//SPE Annual Technical Conference and Exhibition. SPE, 1992: SPE-24825-MS. (1992).

[9] Dayan, A., Stracener, S. M., Clark. P. E. Proppant transport in slick-water fracturing of shale-gas formations[C]//SPE Annual Technical Conference and Exhibition. SPE, 2009: SPE-125068-MS. (2009).

[10] Zhou, D. et al. Experiment and numerical simulation on transportation laws of proppant in major fracture during slick water fracturing. *Oil Drill. Prod. Technol.* **39**(4), 499–508 (2017).

[11] Wang, J. et al. Migration and sedimentation of proppant and its influencing factors in a visual plate fracture model. *Colloids Surf., A* **679**, 132548 (2023).

[12] Gong, F. et al. Dynamics of proppant particle settling within low Reynolds numbers: Roles of particle shape, surface wettability, wall factor and fluid elasticity. *Powder Technol.* **455**, 120741 (2025).

[13] Babcock, R. E., Prokop, C. L., Kehle, R. O. Distribution of propping agents in vertical fractures. Am. Pet. Inst., Publ. ; (United States), 851(CONF-670346), (1967).

[14] Schols, R. S. & Visser, W. Proppant bank build up in a vertical fracture without fluid loss. *SPE* **4834**, 29–30 (1974).

[15] Zhang, T., Guo, J. & Liu, W. CFD Simulation of proppant transportation and settling in water fracture treatments. *J. Southwest Univ. (Sci. Technol. Ed.)* **36**(1), 74–82 (2014).

[16] ZHANG, Z. Experimental and numerical simulation study of proppant delivery in shale gas slickwater fracturing. *Petrochem. Ind. App.* **36**(2), 43–46 (2017).

[17] Liu, C., Li, M., Hao, L. & Guo, T. Numerical simulation of the proppant transportation and setting in the hydraulic fracture. *Daqing Pet. Geol. Dev.* **37**(5), 86–93 (2018).

[18] Chang, O. et al. Physics of proppant transport through hydraulic fracture network. *J. Energy Res. Technol.* **140**(3), 032912 (2018).

[19] Lan, R., Chen, L. & Ran, L. Numerical simulation of the low-density proppant placement in complex fractures. *Pet. Geol. Oilfield Dev. Daqing* **40**(6), 52–61 (2021).

[20] Zhong, A. & Guo, T. Numerical simulation of multi-cluster proppant distribution in horizontal wells. *J. Shenzhen Univ. Sci. Eng.* **39**, 576–583 (2022).

[21] Chengting, L. I. U. et al. Research on the proppant distribution in fractures from channel fracturing. *Henan Sci.* **42**(01), 8–15 (2024).

[22] Tao, Z. et al. Numerical simulation study of proppant transport in complex hydraulic fractures. *Pet. Geol. Recov. Eff.* **31**(03), 123–136 (2024).

[23] HAO, L. CFD numerical simulation of proppant settlement and transport in complex cracks. *Pet. Ind. Appl.* **39**(03), 30–32 (2020).

[24] Wang, J. et al. Migration and settlement laws of proppant in multi-scale fractures during the fracturing in the unconventional reservoirs. *Nat. Gas Ind* **44**(7), 109–119 (2024).

[25] Luo, M. et al. Numerical simulation of proppant transportation and placement in several complex structure fractures of a shale reservoir using a CFD approach. *Gas Sci. Eng.* **118**, 205115 (2023).

[26] Hongzhao, T. et al. Numerical simulation of factors affecting the migration ofhydraulic fracturing proppants in tight gas reservoirs. *Petrochem. Ind. Appl.* **43**(09), 19–23 (2024).

[27] Hu, X. et al. Effect of proppant addition schedule on the proppant distribution in a straight fracture for slickwater treatment. *J. Petrol. Sci. Eng.* **167**, 110–119 (2018).

[28] Junsheng, Z. E. N. G. et al. Numerical simulation of proppant transport law in intersecting fractures. *Fault-Block Oil Gas Field* **28**(05), 691–695 (2021).

[29] Huijuan, G. et al. Investigation on proppant placement in hydraulic fractures based on CFD-DEM. *China Pet. Mach.* **51**(06), 104–111 (2023).

[30] Rui, S. & Zhao, Y. Proppant migration rule during high-speed channel fracturing. *Henan Sci.* **41**(04), 516–523 (2023).

[31] Youan, H. E., Wubin, G. A. O. & Min, L. U. Numerical simulation of fracture propagation of multi-cluster fracturing in horizontal wells[J]. *China Min. Mag.* **30**(5), 200–206 (2021).

[32] Chen, Z. et al. Numerical simulation of proppant transport in transverse fractures of horizontal wells. *Processes* **12**(5), 909–909 (2024).

[33] Li, J. et al. Effect of proppant sizes and injection modes on proppant transportation and distribution in the tortuous fracture model. *Particuology* **84**, 261–280 (2024).

[34] Gidaspow, D., Bezburuah, R., Ding, J. Hydrodynamics of circulating fluidized beds: kinetic theory approach[R]. Illinois Inst. of Tech., Chicago, IL (United States). Dept. of Chemical Engineering. (1990).

[35] Benyahia, S., Syamlal, M. & O’Brien, T. J. Evaluation of boundary conditions used to model dilute, turbulent gas/solids flows in a pipe. *Powder Technol.* **156**(2–3), 62–72 (2005).

